# The Development of Novel Therapies for Chronic Lymphocytic Leukaemia in the Era of Targeted Drugs

**DOI:** 10.3390/jcm14228247

**Published:** 2025-11-20

**Authors:** Tadeusz Robak, Elżbieta Iskierka-Jażdżewska, Anna Puła, Pawel Robak, Bartosz Puła

**Affiliations:** 1 Department of Haematology, Medical University of Lódź, 90-364 Lódź, Polandbartosz.pula@umed.lodz.pl (B.P.); 2 Department of General Haematology and Internal Medicine, Copernicus Memorial, 93-513 Lódź, Poland; 3 Section of Haematology/Oncology, University of Chicago, Chicago, IL 60637, USA; 4 Department of Hematooncology and Internal Medicine, Copernicus Memorial Hospital, 93-513 Lódź, Poland

**Keywords:** BTK degraders, BTK inhibitors, CAR-T, BCL-2 inhibitors, chronic lymphocytic leukaemia, PI3K inhibitors, T-cell engagers

## Abstract

Over the past decade, chronic lymphocytic leukaemia (CLL) treatment has shifted from chemoimmunotherapy to targeted oral agents, predominantly Bruton’s tyrosine kinase inhibitors (BTKis) and the BCL-2 inhibitor venetoclax. These therapies have significantly improved outcomes and are now established as first-line treatment options. However, CLL remains incurable, and resistance or intolerance to both drug classes (double-refractory disease) is an emerging challenge. This has driven the development of novel therapeutic strategies, including non-covalent BTKis such as pirtobrutinib and nemtabrutinib, which retain activity in BTK C481-mutated disease. Next-generation BCL-2 inhibitors (sonrotoclax, lisaftoclax) and BTK degraders are promising in early clinical trials. Immunotherapeutic approaches, such as bispecific T-cell engagers, CD20/CD3 antibodies, and CAR-T cell therapies, provide additional options for high-risk patients. Although PI3K inhibitors remain under investigation, their role is yet to be defined due to safety concerns. Minimal residual disease (MRD)-guided, fixed-duration regimens represent a significant paradigm shift toward personalised treatment and potentially deeper remissions. Ongoing clinical studies are expected to introduce new effective therapies that may further transform the management of CLL in the coming years.

## 1. Introduction

Chronic lymphocytic leukaemia/small lymphocytic lymphoma (CLL/SLL) is the most common form of leukaemia in adults in Europe and North America, with an incidence of 5.1 cases per 100,000 individuals per year [1,2,3]. The disease mainly affects older individuals, with a median age at diagnosis of about 70 years [2,4]. CLL is an indolent, mature lymphoproliferative malignancy characterised by a progressive accumulation of monoclonal B lymphocytes that coexpress CD5, CD19, CD23 and CD20 antigens in peripheral blood (PB), lymph nodes, spleen, and bone marrow (BM). However, some patients present with an atypical CLL immunophenotype, characterised by the absence of expression of one or more surface antigens, most commonly CD5 and CD23 [5]. The cytogenetic characteristics of CLL are heterogeneous, with most patients presenting with deletion 13q, trisomy 12, deletion 11q and notably 17p deletion, which is strongly associated with poor prognosis and resistance to standard therapies [6]. In addition to cytogenetics, *TP53* mutations and the immunoglobulin heavy-chain variable region (*IGHV*) mutational status are key prognostic markers [7]. Patients with *TP53* aberrations and unmutated *IGHV* (um*IGHV*) generally have inferior outcomes, especially when treated with chemoimmunotherapy (CIT) [2].

For many years, the standard treatment for progressive and symptomatic disease in treatment-naïve (TN) and relapsed/refractory (RR) patients has been CIT with cytotoxic drugs (fludarabine, bendamustine, cyclophosphamide, and chlorambucil) and CD20 monoclonal antibodies (rituximab and obinutuzumab). One of the most commonly used regimens is the combination of fludarabine with cyclophosphamide and rituximab (FCR), especially in younger, fit patients [8]. A long-term (19.0-year) follow-up of 300 patients treated with FCR revealed that the median progression-free survival (PFS) was 14.6 years for patients with mutated *IGHV* (m*IGHV*) and 4.2 years for those with um*IGHV* [9]. It was found that FCR remains an acceptable treatment option for selected patients, as only 9% of the m*IGHV* group showed progression beyond 10 years. However, treatment with FCR carries a 1.5–3% risk of developing treatment-related myelodysplastic syndrome (MDS) or acute myeloid leukaemia (AML) [9,10]. This is why, in major guidelines, CIT is no longer recommended for treating CLL patients [11].

Over the past decade, targeted oral therapies—primarily BTKis and the BCL-2 inhibitor venetoclax—have replaced CIT as the standard of care for TN-CLL. Although these agents significantly improve outcomes, CLL remains incurable, and relapse or resistance eventually occurs in most patients [12,13]. As treatment is increasingly guided by targeted drugs rather than CIT, optimal sequencing of therapies has become a major clinical challenge. Patients may relapse after covalent BTKis (cBTKis), venetoclax-based regimens, or combinations thereof, and emerging resistance also occurs following cellular and bispecific antibody therapies. Subsequent treatment decisions depend on prior therapy, response duration, genetic risk factors, and resistance mechanisms. For example, patients relapsing after venetoclax-based fixed-duration therapy may respond to BTKis. At the same time, those progressing on cBTKis may benefit from venetoclax–rituximab or a non-covalent BTKi (ncBTKi) such as pirtobrutinib [4,12,14].

**Figure 1:**
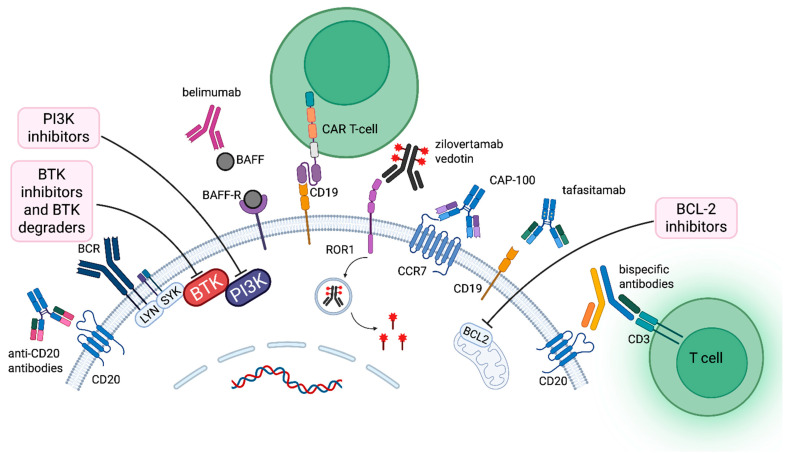
Therapeutic targets and emerging treatment strategies in chronic lymphocytic leukemia. Key membrane-bound proteins and signaling components targeted by novel therapies in CLL, including CD20, CD19, ROR1, BAFF-R, CCR7, BCR, BTK, PI3K, and BCL2. Therapeutic modalities include mAbs, BsAbs, ADCs, CAR-T cells, and small-molecule inhibitors. Abbreviations: ROR1 (Receptor tyrosine kinase-like orphan receptor 1), BAFF-R (B-cell activating factor receptor), CCR7 (C-C motif chemokine receptor 7), BCR (B-cell receptor), BTK (Bruton’s tyrosine kinase), PI3K (Phosphoinositide 3-kinase), BCL2 (B-cell lymphoma 2), mAbs (monoclonal antibodies), BsAbs (bispecific antibodies), ADCs (antibody–drug conjugates). Created in BioRender. Pula, A. (2025) https://BioRender.com/4cl4q8c (accessed on 24 October 2025).

This article discusses the mechanism of action, clinical applications, and safety of novel drugs for CLL (Figure 1). Using Pubmed, Web of Science, and Google Scholar, a literature search was performed for articles published in English. Additional relevant publications were obtained by reviewing the references from the chosen articles.

## 2. BTK Inhibitors

BTKis have become essential drugs in treating CLL [15]. These agents are classified into two types based on their binding mechanism: covalent (irreversible) and non-covalent (reversible). Covalent BTKis approved in CLL include ibrutinib, acalabrutinib, and zanubrutinib. They bind permanently to the C481 residue of the BTK active site. Non-covalent BTKis, such as pirtobrutinib and nemtabrutinib, bind reversibly to BTK and remain effective even if C481S mutations are present (Table 1).

### 2.1. Covalent BTK Inhibitors

Covalent BTKis, including first-generation ibrutinib and second-generation acalabrutinib and zanubrutinib, are established targeted therapies in CLL [15]. In monotherapy they are administered continuously until progression or unacceptable toxicity and have demonstrated superior PFS and OS over CIT, including FCR regimens [18,19,39]. However, their use is limited by adverse events, particularly cardiovascular toxicity such as atrial fibrillation, hypertension, and bleeding [40]. Second-generation inhibitors were developed to improve tolerability while maintaining efficacy [41,42,43]. Clinical trials have confirmed their favourable safety and efficacy profiles in both TN and R/R CLL [24,25,27,28,44,45,46,47].

#### 2.1.1. Ibrutinib

Ibrutinib (Imbruvica, Johnson & Johnson, New Brunswick, NJ, USA) significantly improved PFS and OS compared with chlorambucil or CIT in both TN and RR CLL patients (Table 1) [21,22,23]. The RESONATE trial (phase 3) evaluated RR CLL and demonstrated superior efficacy of ibrutinib over ofatumumab, with a median PFS of 44.1 months versus 8.1 months [48]. The RESONATE-2 trial (phase 3) enrolled TN patients aged ≥65 years and showed that ibrutinib achieved an ORR of 86% and a median PFS of 8.9 years, significantly outperforming chlorambucil [17].

Moreover, two randomised trials (E1912 and FLAIR) demonstrated the superiority of ibrutinib-based therapy over FCR in TN CLL patients [18,19]. In the E1912 study, ibrutinib plus rituximab (IR) significantly improved PFS (HR 0.37; *p* < 0.001) and OS (HR 0.47; *p* = 0.018) compared with FCR, with benefit observed in both m*IGHV* and um*IGHV* subgroups [18]. The IR arm also showed longer OS than the FCR arm (HR, 0.47; *p* = 0.018). The FLAIR trial included TN patients with a median age of 62 years and a WHO performance status of 2 or less [19]. Median PFS had not been reached after a median follow-up of 53 months in the IR arm and 67 months for FCR (HR, 0.44; *p* < 0.0001). However, the IR and FCR arms achieved similar PFS for patients with m*IGHV* (HR 0.64, *p* = 0.15).

The A041202 study in older patients showed that ibrutinib monotherapy and IR yielded superior PFS compared with BR, with no added benefit from rituximab [20]. In *TP53*-aberrant CLL, ibrutinib monotherapy demonstrated a median PFS of 53 months [49]. These findings established continuous BTK inhibition as standard first-line therapy. The FDA approved Ibrutinib monotherapy in 2014 for treating patients with CLL who have received at least one prior therapy and in 2016 for treating TN CLL. The FDA also approved ibrutinib combined with obinutuzumab for TN CLL in 2019 and rituximab in 2020. However, despite its efficacy, long-term therapy with ibrutinib is limited by cardiovascular toxicity [40,50].

#### 2.1.2. The Second-Generation BTK Inhibitors

Second-generation cBTKis, including acalabrutinib and zanubrutinib, were developed to improve tolerability while maintaining or enhancing efficacy compared with first-generation BTKis [41,42,43]. These agents are more selective for BTK, resulting in fewer off-target effects and reduced cardiovascular toxicity.

##### Acalabrutinib

Acalabrutinib (Calquence, AstraZeneca, Cambridge, UK) demonstrated superior efficacy compared with CIT in TN CLL. In the ELEVATE-TN phase 3 trial, acalabrutinib plus obinutuzumab achieved an ORR of 94% and significantly prolonged PFS, with median PFS not reached versus 22.6 months for chlorambucil–obinutuzumab (ChlO) (HR 0.10; *p* < 0.0001) [24]. In RR CLL, the ASCEND trial reported significantly longer PFS for acalabrutinib than idelalisib–rituximab or BR (HR 0.31) [25]. This BTKi also demonstrated non-inferior PFS and lower rates of atrial fibrillation compared with ibrutinib in the ELEVATE-RR trial [26,46]. Acalabrutinib was approved by FDA in monotherapy or combined with obinutuzumab for CLL in 2019.

##### Zanubrutinib

Zanubrutinib (Brukinsa, BGB-3111, BeOne Medicines, Cambridge, MA, USA) has shown improved depth and durability of response compared with standard therapy. In the SEQUOIA trial, zanubrutinib significantly prolonged PFS compared with BR (median PFS not reached vs. 44.1 months; HR 0.29; *p* = 0.0001) [28]. Moreover, in the ALPINE trial, zanubrutinib demonstrated superior PFS (24-month PFS 78.4% vs. 65.9%; HR 0.65) and higher ORR (83.5% vs. 74.2%) compared with ibrutinib in RR CLL, with fewer cardiac adverse events [29]. In January 2023, the FDA approved zanubrutinib for the first-line treatment of CLL/SLL.

Both acalabrutinib and zanubrutinib are now preferred over first-generation BTKis due to improved safety profiles and sustained efficacy in high-risk CLL. Several other next-generation cBTKis, including orelabrutinib and tirabrutinib, are currently being investigated for CLL and other B-cell malignancies [15].

##### Orelabrutinib

Orelabrutinib (ICP-022, Hibruka, InnoCare, Beijing, China) is a next-generation cBTKi that demonstrated in vitro greater selectivity against BTK than ibrutinib, with a more than 90% BTK inhibition rate [31]. A phase II study reported its activity on B-cell malignancies, including 80 patients with RR CLL. Treatment achieved a 93.8% ORR. At a median follow-up of 32.3 months, the median PFS had not been achieved, while the 30-month PFS and OS rates were 70.9% (95% CI, 59.5–79.6) and 81.3% (95% CI, 70.8–88.2), respectively [30]. Essentially, no cases of AF or severe hypertension were observed. Orelabrutinib was approved in China for previously treated CLL/SLL and is currently authorised for that indication.

##### Tirabrutinib

Tirabrutinib (Velexbru, ONO/GS-4059, Ono Pharmaceutical Co., Ltd., Osaka, Japan) is another next-generation cBTKi, more selective than ibrutinib, and is being investigated in B-cell malignancies. In a phase 1 trial, tirabrutinib was well tolerated in RR mature B-cell lymphoid malignancies [51]. This drug was studied in CLL with entospletinib, with or without obinutuzumab, and demonstrated therapeutic activity with good tolerability [32]. This BTKi demonstrates superior blood–brain barrier penetration compared with ibrutinib and may represent a promising therapeutic option for CLL patients with central nervous system involvement [33]. It has received approval in Japan for treating primary central nervous system lymphoma. Reversible non-covalent BTKis, pirtobrutinib and nemtabrutinib, do not require C481 for their antineoplastic activity and can be effective in patients refractory to covalent BTK inhibitors.

### 2.2. Non-Covalent BTK Inhibitors

Non-covalent BTKis are next-generation, reversible agents that effectively inhibit BCR signalling in both TN and ibrutinib-refractory CLL. Unlike cBTKis, they do not depend on binding to the C481 residue, which allows them to maintain activity in patients who have developed resistance due to C481 mutations. Acquired mutations in BTK or its downstream mediator PLCγ2 have been identified in most cases resistant to ibrutinib, acalabrutinib, and zanubrutinib [52,53,54]. Non-covalent BTKis maintain efficacy irrespective of the C481S mutation [34,35]. Pirtobrutinib (LOXO-305) and nemtabrutinib are the most advanced ncBTKis tested in clinical trials.

#### 2.2.1. Pirtobrutinib

Pirtobrutinib (LOXO-305, Jaypirca, Eli Lilly, Indianapolis, IN, USA) received FDA approval in December 2023 to treat patients with CLL who had previously received both a cBTKis and a BCL-2 inhibitor [34]. This approval was based on data from the phase 1/2 BRUIN trial, which enrolled patients with heavily pretreated CLL or SLL who had progressed after cBTK exposure. [34]. However, the FDA approval specifically relates to the subgroup that had received both a BTKi and a BCL-2 inhibitor. In this study, pirtobrutinib demonstrated meaningful clinical activity, achieving an ORR of 73.3% and a median PFS of 19.6 months among 247 patients with RR CLL/SLL [44]. The treatment was generally well tolerated; the most common adverse events were infections (71.0%), bleeding (42.6%), and neutropenia (32.5%) [44]. Other observed adverse events included hypertension (14.2%), atrial fibrillation or flutter (3.8%), and major haemorrhage (2.2%) [34].

Several ongoing clinical trials are further evaluating pirtobrutinib in CLL and SLL. A phase 2 trial assesses the safety and efficacy of three dosing regimens in patients with RR CLL or SLL who have received one to three prior lines of therapy, including cBTKi, with study completion anticipated in 2028 (#NCT06466122). Another phase 1/2 trial (#NCT03740529) is investigating pirtobrutinib in patients with CLL, SLL, and non-Hodgkin lymphoma (NHL) who are refractory or intolerant to standard-of-care therapies, with an estimated completion date in 2028.

In phase 3 studies, pirtobrutinib is being compared with ibrutinib in patients with CLL/SLL carrying del (17p) (BRUIN CLL-314; #NCT05254743), with BR in TN patients (BRUIN CLL-313; #NCT05023980), and with investigator’s choice of idelalisib plus rituximab or BR in previously treated patients (BRUIN CLL-321; #NCT04666038). Additionally, pirtobrutinib is being evaluated in combination regimens, including phase 2 trials with venetoclax in patients resistant to cBTKis (#NCT06466122) and in TN patients with MRD assessment (#NCT05677919). Further studies include pirtobrutinib combined with obinutuzumab in TN CLL (#NCT06333262) and a phase 3 trial assessing the triplet regimen of pirtobrutinib, venetoclax, and rituximab (PVR) versus venetoclax and rituximab (VR) in RR CLL/SLL (BRUIN CLL-322; #NCT04965493). A time-limited triplet combination of pirtobrutinib, venetoclax, and obinutuzumab is also being investigated in TN CLL and in patients with Richter transformation (RT) (#NCT05536349).

Overall, the expanding body of clinical evidence supports pirtobrutinib as a highly promising therapeutic option for patients with CLL across multiple lines of therapy, particularly those with resistance to cBTKis.

#### 2.2.2. Nemtabrutinib

Nemtabrutinib (MK-1026, ARQ-531, Merck, Rahway, NJ, USA) is another ncBTKi with a mechanism similar to pirtobrutinib, which reversibly inhibits both wild-type and C481-mutated BTK. Nemtabrutinib was tested in 47 patients with RR disease, including 29 with CLL, 17 with B-cell NHL, and one with Waldenström macroglobulinemia (WM), at doses ranging from 5 to 75 mg once daily in 28-day cycles [35]. An ORR was noted in 75% of CLL patients receiving the compound at a daily dose of 65 mg. Grade ≥ 3 treatment-emergent adverse events (TAES) were observed in 37 patients (89%), most frequently neutropenia (23.4%), febrile neutropenia (14.9%), and pneumonia (14.9%) [36]. Several late-phase clinical trials are ongoing to evaluate nemtabrutinib in CLL/SLL. The phase 2 BELLWAVE-003 trial (#NCT04728893) is assessing the efficacy and safety of nemtabrutinib in various B-cell malignancies, including CLL/SLL, at the recommended phase 2 dose, with study completion expected in 2027. In parallel, the phase 3 open-label randomized BELLWAVE-011 trial (#NCT06136559) is comparing nemtabrutinib (65 mg once daily) with the investigator’s choice of ibrutinib or acalabrutinib in TN patients with CLL/SLL. Results from this study are not yet available.

Further, nemtabrutinib is being investigated in multiple phase 3 studies across different lines of therapy. The BELLWAVE-008 trial (NCT05624554) compares nemtabrutinib with CIT in TN CLL/SLL patients without *TP53* aberrations. The BELLWAVE-010 trial (NCT05947851) evaluates nemtabrutinib in combination with venetoclax versus venetoclax plus rituximab (VR) in patients with RR CLL/SLL as second-line therapy, with an estimated study completion in 2033. In addition, NCT05458297 (MK-2140-006) is investigating zilovertamab vedotin alone and in combination with nemtabrutinib in patients with aggressive and indolent B-cell malignancies, including CLL, with completion expected in 2027. Preclinical data support combination strategies. In a CLL mouse model, nemtabrutinib combined with venetoclax significantly prolonged survival compared with ibrutinib plus venetoclax, highlighting its potential synergy and supporting clinical evaluation of this combination [15,55].

#### 2.2.3. Rocbrutinib

Covalent BTKi and nc BTKi are effective in CLL treatment; however, second-site T474 gatekeeper mutations (GM) are commonly observed in patients resistant to ncBTKi such as pirtobrutinib [38]. Rocbrutinib,(LP-168, Hansoh Pharma (Lianyungang, Jiangsu, China) and Lupeng Pharma (Guangzhou, Guangdong, China) is a selective next-generation BTKi that irreversibly targets wild-type BTK, reversibly targets C481 mutant BTK (C481S, C481F, and C481R), and irreversibly targets other non-C481 mutations, such as the GM T474I, with preclinical activity in C481 and T474 mutants and clinical activity in RR CLL. In a phase 1 trial, 10 patients who had previously received cBTKi and ncBTKi treatment were subjected to ibrutinib treatment. With a median follow-up of 14 months (range 2.6–20.1), responses were observed in 9 patients. The ORR across all dose levels was 77.8% (7/9), with all being PR or PR-L [38].

## 3. BTK Degraders

Proteolysis-targeting chimaeras (PROTACS) can potentially overcome acquired resistance to BTKis in B-cell malignancies. They offer improved target selectivity, rapid and sustained BTK depletion, and greater potency in BTK inhibition degradation [56,57]. BTK degraders (BTKdeg) are targeted protein degraders that induce the degradation of BTK [58]. BTKdeg can potentially prevent signal transduction from BCR receptor and may be clinically helpful in BTKi intolerance or resistance in CLL. BGB-16673, Nx-2127, Nx-5948, NRX-0492, HZ-Q1060, ABBV-101, and AC676 have shown significant BTK degradation in preclinical studies and early clinical trials (Table 2) [57]. These BTKis have demonstrated favourable safety profiles and are considered novel therapeutic agents for patients with BTKi-resistant diseases [59].

### 3.1. BGB-16673

BGB-16673 (BeOne Medicines, Cambridge, MA, USA) is a bivalent BTKdeg that induces BTK degradation by specifically binding to BTK and the E3 ligase. It exhibits a unique on-target resistant mutation profile and could overcome many BTK resistance mutations. BGB-16673 showed promising preclinical BTK degradation activity. In the phase 1 study (CaDAnCe-101), BGB-16673 was safe and well-tolerated in the heavily pretreated population of patients with RR CLL/SLL [60]. In 49 response-evaluable patients, BGB-16673 showed significant antitumour activity, including in patients with BTKi–resistant mutations and those previously exposed to cBTK, ncBTK, and BCL2 inhibitors. The median time to first response was 2.8 months (2.6–8.3 months). ORR was 77.6% (38/49) and CR/CRi 4.1% (2/49). The best response was observed in patients treated with 200 mg administered orally once daily in 28-day cycles (ORR 93.8%). Promising activity was also seen in RT, with an ORR of 58.3% (7/12) and a CR of 8.3% (1/12). A Phase 1b/2 study of BGB-16673 in combination with sonrotoclax, zanubrutinib, mosunetuzumab, or glofitamab in patients with RR B-cell malignancies is ongoing (#NCT06634589). A phase 3 study evaluating the safety and efficacy of BGB-16673 compared to pirtobrutinib in adults with RR CLL/SLL is underway (#NCT06973187). In parallel, the phase 3 study (CaDAnCe-302) of BGB-16673 compared to the investigator’s choice in participants with CLL/SLL previously exposed to both BTK and BCL2 inhibitors (#NCT06846671) was recently initiated. Two phase 1/2 open-label, dose-escalation, and dose-expansion clinical trials test BGB-16673 in patients with CLL and other B-cell malignancies (#NCT05294731 and #NCT05006716). The studies are expected to be completed in 2027 and 2028, respectively.

### 3.2. *Bexobrutideg*

*Bexobrutideg* (*NX-5948*, Nurix Therapeutics Inc., San Francisco, CA, USA) is a chimeric targeting molecule (CTM) that induces BTK protein degradation by the cereblon E3 ligase (CRBN) complex without degradation of other cereblon neo-substrates. In a phase 1a/b study, 87 patients with different B-cell malignancies, including 34 patients with CLL, were included [61]. Prior therapies included BTKi (97.1%), pirtobrutinib (23.5%), BCL-2 inhibitors (91.2%), BTKi + BCL2 inhibitors (88.2%), chemotherapy/CIT (79.4%), PI3K inhibitors (32.4%), and CAR-T (5.9%). Bexobrutideg was well tolerated across all tested doses, consistent with previously reported safety profiles. The most common TEAEs were purpura/contusions (44.1%), thrombocytopenia (23.5%), petechiae (29.4%), fatigue (20.6%), and neutropenia (17.6%). There was no new onset of atrial fibrillation/flutter or hypertension. In 30 response-evaluable patients with CLL, the ORR was 76.7%, with 22 responses ongoing at data cut-off. Additionally, 29 out of 34 patients remained on treatment.

### 3.3. NX-2127

NX-2127 (Nurix Therapeutics, Inc., San Francisco, CA, USA) is a BTKdeg with concomitant immunomodulatory activity mediating the degradation of transcription factors IKZF1 and IKZF3 through molecular glue interactions with the cereblon E3 ubiquitin ligase complex [62]. Treatment with NX-2127 induced >80% degradation of BTK in CLL patients. NX-2127 degrades common BTK resistance mutants, including BTK^C481S^ [63]. In the first-in-human clinical trial, deep and sustained degradation of BTK was achieved in daily oral dosing at 100 mg [64]. In heavily pretreated patients with RR CLL and NHL, encouraging and sustained responses were observed with a manageable safety profile. The safety and anticancer activity of NX-2127 are being assessed in a first-in-human phase 1a/1b clinical trial involving patients with R/R B-cell malignancies (#NCT04830137). The study is expected to be completed in 2026.

### 3.4. AC676

AC676 (Accutar Biotech., Cranbury, NJ, USA) is an orally bioavailable BTKdeg designed to treat B-cell malignancies [65]. It was designed as a chimeric degrader to specifically target and degrade BTK using a proprietary Protein–Protein Interaction Targeting Chimeras (PPI-TAC) platform by Accutar Biotech. AC676 recruits BTK by linking a BTK ligand to the cereblon E3-ligase recruiting ligand, bringing it into proximity with cereblon, which then induces ubiquitination and degradation of BTK proteins, including those with resistance mutations such as C481 and kinase-dead variants L528. As a result, AC676 may be an effective treatment for CLL patients progressing on both cBTKis and ncBTKis. However, AC676 does not degrade cereblon neo-substrates, which is expected to limit toxicity, particularly neutropenia, as a consequence of the treatment. AC676-001 is being investigated in a phase 1, dose-escalation study in patients with RR B-cell malignancies (ClinicalTrials.gov ID NCT05780034). AC676 is administered as a single agent orally once daily on a 28-day cycle, with doses ranging from 50 mg to 600 mg [65]. The study is estimated to be completed in 2026.

### 3.5. NRX-0492

NRX-0492 (Nurix Therapeutics Inc., San Francisco, CA, USA) is a targeted protein degrader that binds a non-covalent BTK-binding domain to an adaptor protein, cereblon of the E3 ubiquitin ligase complex. NRX-0492 demonstrated activity against WT BTK and C481 mutants in CLL in vitro and in vivo [58]. In PDX CLL in vivo models, NRX-0492–induced BTK degradation and inhibited CLL cell activation, proliferation, and expansion. Clinical studies testing NRX-0492 have been initiated (#NCT04830137).

### 3.6. HZ-Q1060 and HZ-Q1070

HZ-Q1060 and HZ-Q1070 (Hangzhou HealZen Therapeutics Co., Hangzhou, China) were developed as novel BTK-PROTAC agents within the DaTProD^®^ platform and have been validated as promising candidates for further clinical development [69]. HZ-Q1060 can selectively degrade BTK in lymphoma cell lines, human PBMCS, and intracellular mutant BTK-C481S protein. The drug was also investigated in the subcutaneously transplanted tumour model of OCI-LY10 mice, where tumour growth was entirely inhibited by HZ-Q1060 when administered orally every day for 14 days. HZ-Q1070 is another promising BTKdeg with excellent pharmacokinetic properties for clinical use development [67]. It exhibited tumour growth inhibition in cell experiments and a mouse tumour model. Moreover, HZ-Q1070 avoided degradation of Aiolos and Ikaros, essential for NK cell activity [66]. A phase I study of HZ-Q1070 in patients with RR B-cell malignancies, including CLL/SLL, WM, mantle cell lymphoma (MCL), follicular lymphoma (FL), marginal zone lymphoma (MZL), diffuse large B-cell lymphoma (DLBCL) and primary central nervous system lymphoma (PCNSL), is in progress (#CTR20240471).

### 3.7. ABBV-101

ABBV-101 (AbbVie Inc., Chicago, IL, USA) is a reversible BTKdeg with high selectivity and activity against BTK wild type and multiple therapy-resistant mutant forms in preclinical studies. ABBV-101 has shown efficacy in vitro and in vivo in BTK-WT and cBTKi-resistant (BTK-C481S) systemic mouse CLL and human DLBCL models. Moreover, ABBV-101 induces complete tumour regressions in multiple non-GCB DLBCL PDX models, showing deeper and longer responses than covalent and reversible inhibitors. Significantly, the BCL-2 inhibitor enhances the efficacy of ABBV-101 in CLL and DLBCL models. ABBV-101 is investigated in a phase 1, open-label, multicenter study in patients with B-cell lymphoid malignancies, including CLL (#NCT05753501) [68].

## 4. BCL-2 Inhibitors

BCL-2 inhibitors target the BCL-2 protein, which is overexpressed in CLL and other haematological malignancies. Venetoclax is the first BCL-2 inhibitor approved for the treatment of CLL. Second-generation BCL-2 inhibitors, sonrotoclax, lisaftoclax, mesutoclax, and ABBV-453 are currently under investigation in CLL (Table 3).

### 4.1. Venetoclax

Venetoclax (formerly ABT-199, Venclyxto, AbbVie Inc., Chicago, IL, USA) is a first-in-class BCL-2 inhibitor that blocks BCL-2 signalling and induces apoptosis [79]. Single-drug venetoclax treatment was initially approved for CLL patients with 17p deletion in 2016 [80]. It should be administered continuously when given as a single agent. The more commonly used option is time-limited treatment with venetoclax combined with CD20 antibodies or BTKis [81,82,83,84]. Such regimens can induce undetectable MRD at the end of treatment, which is an independent predictor of improved OS in patients with TN or RR CLL/SLL.

In the phase 3 MURANO trial, treatment with fixed-duration venetoclax plus rituximab (VR) for up to 2 years demonstrated durable remissions in patients with RR CLL [70]. VR resulted in superior PFS and OS compared with BR. In the final analysis, with a median follow-up of 7 years, the median PFS was 54.7 months with VR and 17.0 months with BR. Among 25 patients retreated with VR, the median PFS was 23 months, and the best ORR was 72% [70].

In 2019, the FDA approved venetoclax in combination with obinutuzumab (VenO) for TN CLL based on the CLL14 phase 3 randomised trial, comparing VenO with ChlO in 432 TN patients with coexisting conditions [84]. Significantly higher PFS was observed at 24 months for patients who received VenO (82.2%) compared with those who received ChlO (64.1%) (HR 0.33; *p* < 0.0001). VenO was also more beneficial in high-risk subgroups, including patients with *TP53* deletion and those with um*IGHV*. A six-year follow-up confirmed that fixed-duration VenO offers greater long-term PFS than ChlO treatment [71]. At a median observation time of 76.4 months, median PFS was 76.2 months for VenO and 36.4 months for ChlO (HR, 0.40; *p* < 0.0001). Additionally, over 60% of patients treated with VenO did not require second-line treatment. In the VenO arm, del (17p) and um*IGHV* were independent prognostic factors for shorter PFS. These findings confirm that the fixed-duration VenO regimen is an effective option for previously untreated patients with CLL.

### 4.2. Combination of BTKi and Venetoclax

The efficacy and tolerability of venetoclax combined with BTK inhibitors have also been established, offering another fixed-duration alternative for patients with CLL [39,85,86,87,88,89,90]. In the CAPTIVATE phase 2 study, 159 patients aged ≤70 years with previously untreated CLL received three one-month cycles of ibrutinib, followed by 12 cycles of ibrutinib plus venetoclax (I + V) [85]. The CR rate was 55%, with the best undetectable MRD rates being 77% in PB and 60% in BM; the 24-month PFS and OS rates were 95% and 98%, respectively. The most common grade 3 or higher AEs were neutropenia (33%) and hypertension (6%).

The phase 3 GLOW study compared I + V with ChlO in 211 previously untreated patients with CLL [86,87,88]. The patients’ median age was 71 years, and the median CIRS score was 9. At a median follow-up of 27.7 months, PFS events were observed in 22 I + V patients and 67 ChlO patients. At a 54-month follow-up, the median follow-up PFS rates were 65.8% for I + V and 19.1% for ChlO. In the I + V arm, patients with um*IGHV* demonstrated shorter PFS (58%) than those with m*IGHV* (90%). At 54 months, the I + V arm also achieved better estimated OS (84.5%) than the ChlO arm (63.1%).

The GAIA/CLL13 study compared CIT with FCR or BR with VR, VenO, or VenO plus ibrutinib [39]. At month 15, the VenO (86.5%) and VenO plus ibrutinib (92.2%) groups were significantly more likely to present undetectable MRD than the FCR or BR group (52.0%). No significant difference was noted between the VR group and the CIT arm (57%). PFS at three years was higher in the VenO plus ibrutinib group (90.5%) and VenO group (87.7%) than in the CIT group (75.5%) or the VR group (80.8%) [39].

The FLAIR phase 3 randomised trial compared I + V and ibrutinib with FCR in TN CLL [91]. The duration of I + V treatment was related to MRD status: at a median of 43.7 months, disease progression was observed in 12 patients in the I + V arm and 75 patients in the FCR arm (*p* < 0.001). Nine deaths were noted in the I + V group and 25 in the FCR group. At three years, 58.0% of the patients in the I + V arm had stopped therapy due to undetectable MRD. These findings support using VenO or I + V combinations in the first-line treatment of patients with CLL.

Acalabrutinib plus venetoclax, with or without obinutuzumab, was also compared with CIT in previously untreated patients in the AMPLIFY phase 3 study [92]. Patients were randomly assigned to receive acalabrutinib plus venetoclax, acalabrutinib plus venetoclax and obinutuzumab, or CIT with FCR or BR. The estimated 36-month PFS rates at a median follow-up of 40.8 months were 76.5%, 83.1%, and 66.5%, respectively (*p* = 0.004). The estimated 36-month OS rates were 94.1%, 87.7%, and 85.9%.

The arm D SEQUOIA study evaluated zanubrutinib in combination with venetoclax in patients with TN CLL/SLL with del (17p) [93]. The early result included 35 of approximately 80 planned patients with a median follow-up of 9.7 months. The initial efficacy assessment three months after starting zanubrutinib indicated an ORR of 96.8% (30/31 patients). Zanubrutinib plus venetoclax was well tolerated, and no new safety signals were reported.

### 4.3. Second-Generation BCL-2 Inhibitors

New BCL-2 inhibitors active in lymphoid neoplasms are currently under investigation. The most advanced in clinical trials include sonrotoclax (BGB-11417), lisaftoclax (APG-2575), ABBV-453, and mesutoclax (ICP-248) [74,94,95,96,97].

#### 4.3.1. Sonrotoclax

Sonrotoclax (BGB-11417, BeOne Medicines, Cambridge, MA, USA) is a more selective and more pharmacologically potent inhibitor of BCL-2 than venetoclax. Moreover, it has a shorter half-life and does not accumulate in the body observed [94]. Moreover, sonrotoclax demonstrated significantly greater efficacy in xenograft mouse models and superior activity compared to venetoclax in vitro, in inhibiting both WT Bcl-2 and the G101V mutant. Currently, sonrotoclax is undergoing clinical evaluation as a monotherapy and in combination with other drugs. Sonrotoclax was investigated in combination with zanubrutinib in TN patients with CLL [73]. Among 108 evaluated patients, the ORR was 100% (CR 41% for the 160 mg dose and 42% for the 320 mg dose). No progression was observed at the 320 mg dose. Treatment was well tolerated, with no cases of laboratory or clinical TLS, and only one patient discontinued treatment due to a TEAE. A phase 3 study (CELESTIAL-TNCLL, BGB-11417-301) with this combination is currently ongoing (#NCT06073821). A single-arm, open-label, multicentre phase 2 clinical trial is assessing the efficacy of sonrotoclax in 100 adult patients with RR CLL/SLL in China. The estimated study completion date is in 2027. In parallel, sonrotoclax is also being studied with zanubrutinib in three clinical trials (#NCT06637501, #NCT06073821, and #NCT06367374).

#### 4.3.2. Lisaftoclax

Lisaftoclax (APG-2575, Ascentage Pharma, Suzhou, China, and Rockville, MD, USA) is a novel, specific BCL-2 inhibitor active in patients with TN and RR CLL/SLL, including those with 17p deletion and progressive disease after BTKi therapy [98]. Lisaftoclax used alone or in combination with acalabrutinib or rituximab had a manageable safety profile and was active in patients with previously untreated or RR CLL/SLL. In the first-in-human open-label trial in patients with RR CLL, lisaftoclax treatment was associated with an ORR of 63.6% and a rapid reduction in absolute lymphocyte count, even at the lowest doses, with response on the first day of dosing. In patients with CLL/SLL and 17p deletion, an ORR was 66.7% [74]. Lisaftoclax combined with acalabrutinib or rituximab also had a manageable safety profile and was active in patients with previously untreated or RR CLL/SLL [99]. Several ongoing studies evaluate the efficacy and safety profiles of lisaftoclax in CLL and other haematological malignancies. A global multicentre, open-label, randomised, registrational phase 3 clinical trial is currently recruiting approximately 400 participants with CLL who have been previously treated with BTKis to assess the safety and efficacy of lisaftoclax in combination with BTKis. The estimated study completion date is 2027, with an anticipated primary completion date in October 2025 (#NCT06104566). Another global, multicenter, randomised, open-label, phase 3 confirmatory trial is also recruiting participants with newly diagnosed CLL to investigate the safety and efficacy of lisaftoclax in combination with acalabrutinib. This study is expected to be completed in 2028 (#NCT0502404528).

#### 4.3.3. Surzetoclax

Surzetoclax (ABBV-453, AbbVie Inc., Chicago, IL, USA) is a next-generation BCL-2 inhibitor [93]. The activity of ABBV-453 as a monotherapy was investigated in an in vivo subcutaneous xenograft model of CLL. The study demonstrated superior growth inhibition of the RS4;11 xenograft compared with sonrotoclax or lisaftoclax at equivalent doses and schedules [75]. ABBV-453 has the potential to be the best-in-class next-generation BCL-inhibitor and is actively being investigated in phase 1 clinical trials in RR CLL (#NCT06291220) and RR multiple myeloma (#NCT05308654).

#### 4.3.4. LOXO-338

LOXO-338 (Eli Lilly, Indianapolis, IN, USA) is a novel inhibitor of BCL-2, designed to inhibit Bcl-2 over Bcl-xL selectively and to avoid dose-limiting thrombocytopenia associated with Bcl-xL inhibition [94]. A phase 1 clinical trial tests the safety and effectiveness of LOXO-338 in patients with advanced B-cell malignancies, including CLL, who have already received standard treatment therapy (#NCT05024045) [76]. In total, 27 patients were enrolled, including 10 with CLL/SLL and 17 with NHL. The ORR was 19%, and disease was controlled in 67%. TEAEs were reported in 23 patients (85%), including anaemia (22%) and fatigue (22%). Serious TEAEs and TLS were not observed [76].

#### 4.3.5. Mesutoclax

Mesutoclax (ICP-248, InnoCare Pharma, Beijing, China) is a novel, orally bioavailable BCL-2 selective inhibitor. In a phase 1 study, 68 patients were enrolled in the dose escalation and expansion study (#NCT05728658) [78]. Among the BTK-naïve patients, the ORR for RR CLL/SLL and RR MCL patients was 100%, including CR 14.3% for RR CLL/SLL and 71.4% for RR MCL. In 43% of MCL patients, undetectable MRD was reported. Among the BTK-treated patients, the ORR for RR CLL/SLL and RR MCL patients was 100% and 78.9%, respectively, and the CRR were 30.0% and 26.3%, respectively, of which uMRD was reported in 20% of CLL/SLL and 16% of MCL patients. The median PFS of RR MCL patients who had previously received treatment with BTKis was 8.3 months. The PFS was not reached among BTK-naïve RR CLL/SLL and RR MCL patients and BTK-treated RR CLL/SLL patients. In another dose optimisation study in patients with TN CLL/SLL, mesutoclax plus orelabrutinib treatment is guided by MRD status (#NCT06378138). The preliminary efficacy and safety data of mesutoclax + orelabrutinib for CLL/SLL were recently presented (ICP-CL-01203/#NCT06378138) [77]. The patients are randomly assigned 1:1 for treatment with mesutoclax (100 mg or 125 mg once daily, cycles 3–14) after two cycles of orelabrutinib (150 mg once daily, cycles 1–17). Mesutoclax was started with a 5-week ramp-up schedule to the target doses (5/10 mg, 25 mg, 50 mg, 75 mg, 100/125 mg) to control TLS. Orelabrutinib is administered continuously for undetectable MRD at cycle 17. In the first report, 42 patients were included. The median treatment duration was 7.5 months; all patients were on treatment. The ORR was 100%, and an early high uMRD rate at 12 weeks of target therapy was observed in patients receiving the 125 mg dose. No progression was seen in either cohort. No clinical or laboratory TLS occurred. The most common TEAEs were neutropenia, thrombocytopenia, and upper respiratory tract infections. A phase 3 registration study is planned to evaluate the efficacy and safety of the combination of orelabrutinib and mesutoclax at 125 mg QD.

## 5. PI3K Inhibitors

Phosphoinositide 3-kinases (PI3Ks) signalling regulates growth, survival, differentiation, activation, and apoptosis of B and T cells. PI3K inhibitors have been extensively studied in many cancers, including B-cell malignancies and CLL [97,98]. For RR CLL patients with limited options, PI3K inhibitors like idelalisib combined with rituximab may be considered [100]. However, the toxicity of this treatment is high [100,101]. The FDA approved three PI3K inhibitors, idelalisib, duvelisib and umbralisib, to treat CLL and/or other lymphoid malignancies (Table 4).

### 5.1. Idelalisib

Idelalisib (GS-1101, CAL-101, Zydelig, Gilead Sciences, Inc., Foster City, CA, USA) was the first-in-class PI3Kδ inhibitor registered for treating RR CLL. In a multicentre, phase 3 randomised trial conducted in patients with RR CLL, idelalisib and rituximab were compared to rituximab alone [102]. This study was stopped early due to the advantage of the idelalisib-treated arm over the control arm. After a median follow-up of 18 months, PFS was 20.3 months in the rituximab plus idelalisib arm and 6.5 months in the rituximab arm. However, the toxicity of this treatment was high [122]. Idelalisib causes fatigue, diarrhoea, nausea, chills, and skin changes, which are mostly low-grade in severity. Also, like other PI3K inhibitors, idelalisib can cause hepatotoxicity, diarrhoea, colitis, skin changes, and infections.

### 5.2. Duvelisib

Duvelisib (IPI-145, INK1197, Copiktra, Secura Bio, Inc., Las Vegas, NV, USA) is a selective dual inhibitor of Pi3kδγ. In the phase 2 DYNAMO trial (#NCT01476657), among 28 patients with CLL/SLL refractory to rituximab and chemotherapy or radioimmunotherapy, the ORR was 68%, and all were PR [103]. In a phase 3 DUO trial (#NCT02004522), duvelisib was compared with ofatumumab in patients with RR CLL/SLL [123]. The PFS was significantly longer in patients treated with duvelisib (13.3 months) than in the ofatumumab arm (9.9 months). The ORR was also higher in the duvelisib group (74% vs. 45%) [124]. The most common non-haematological adverse events in patients treated with duvelisib were diarrhoea (51%), pyrexia (29%), nausea (23%), cough (21%) and colitis (13%). Duvelisib was approved by the FDA in 2018 and the EMA in 2021 for treating RR CLL/SLL patients after at least two prior therapies.

### 5.3. Umbralisib

Umbralisib (Ukoniq, TG Therapeutics, Morrisville, NC, USA) is a next-generation inhibitor of PI3Kδ and casein kinase-1ε (CK1ε) with a lower incidence of autoimmune complications [107,108,125]. In a phase 2 study, umbralisib was evaluated in 51 patients with CLL who were intolerant to prior BTKis (*n* = 44) or PI3Kδis (*n* = 7), and 48 were evaluable for response [106]. Median PFS was 23.5 months, and ORR was 44%. The most common adverse events were rash (27%), arthralgia (18%), and atrial fibrillation (16%), while the most common grade ≥ 3 AEs were neutropenia (18%), leukocytosis (14%), thrombocytopenia (12%), pneumonia (12%), and diarrhoea (8%). Umbralisib received fast-track FDA approval for the treatment of CLL in combination with an anti-CD20 antibody in 2020. However, the developer withdrew this combination for the treatment of CLL based on updated OS data from the UNITY-CLL trial in 2022.

### 5.4. Novel PI3K Inhibitors

Novel PI3K inhibitors investigated in patients with CLL include zandelisib, parsaclisib, BGB-10188, tenalisib, ACP-319, HMPL-689, SHC014748M, TQ-B3525, and linperlisib.

#### 5.4.1. Zandelisib

Zandelisib (PWT143, ME-401, Mei Pharma, San Diego, CA, USA) is a selective, potent PI3Kδ inhibitor with prolonged target inhibition and a high volume of distribution, indicating high tissue exposure [109]. The drug is in clinical development for treating patients with B-cell lymphoid malignancies, including RR CLL and B-cell NHL. In a phase 1 study of zandelisib, used alone or in combination with rituximab, durable objective responses were observed in RR indolent B-cell malignancies (FL, CLL/SLL, MZL and DLBCL; #NCT02914938) [111]. The ORR was 83%, including 89% in CLL/SLL (100% in monotherapy and 83% in the combination group), with a median DOR not reached. The drug was well tolerated, with no safety differences observed between the monotherapy and rituximab combination groups. A phase II study evaluated zandelisib in RR FL and MZL [110]. The ORR was 75.4% and the CR was 24.6%. At least one Grade ≥ 3 TEAE was reported in 55.7% of patients, including transaminase elevation (8.2%), cutaneous reactions (3.3%), and diarrhoea, enterocolitis, and lung infections (1.6% each). A phase 2 trial has also been initiated combining zandelisib with rituximab and venetoclax in patients with RR CLL (CORAL) (#NCT05209308).

#### 5.4.2. Parsaclisib

Parsaclisib (INCB50465, IBI-376, Incyte, Wilmington, DE, USA) is a highly selective PI3Kδ inhibitor. In the first-in-human phase 1/2 CITADEL-101 study, parsaclisib demonstrated antitumor activity in RR B-cell NHL (#NCT02018861) [113]. The drug was studied alone or in combination with itacitinib (Janus kinase 1 inhibitor) or CIT with rituximab, ifosfamide, carboplatin, and etoposide in patients with RR B-cell malignancies (#NCT02018861) [113]. Parsaclisib demonstrated durable responses and a manageable safety profile in RR MZL [112]. Parsaclisib is currently under evaluation in combination with the anti-CD19 monoclonal antibody tafasitamab in patients with RR CLL and RR NHL as part of a phase 1/2 study (#NCT04809467).

#### 5.4.3. BGB-10188

BGB-10188 (BeOne Medicines, Cambridge, MA, USA) is a highly selective inhibitor of PI3Kδ, with an improved safety profile compared with other PI3Kis. BGB-10188 is being investigated as a monotherapy and in combination with zanubrutinib or PD-1 antibody tislelizumab in patients with CLL and other B-cell NHLs in a phase 1/2 clinical trial (#NCT04282018) [126].

#### 5.4.4. Tenalisib

Tenalisib (GDC-0032, RP6530, Rhizen Pharmaceuticals SA, Basel, Switzerland) is a dual PI3K δ/γ inhibitor with demonstrated antitumor activity in a T cell leukaemia xenograft model in mice and in patient-derived primary cutaneous T cell lymphoma (CTCL) cells [115]. A phase 2 study has also been initiated investigating the efficacy and safety of tenalisib in patients with RR CLL after at least one prior therapy (#NCT04204057).

#### 5.4.5. ACP-319

ACP-319 (AMG 319, Acerta Pharma BV, Oss, The Netherlands/AstraZeneca, Cambridge, UK) is a novel selective PI3Kδ inhibitor with promising results in early preclinical and clinical studies. The first human study of ACP-319 evaluated the drug’s safety, tolerability, and pharmacokinetics in 28 heavily pretreated patients with RR CLL (*n* = 25) and RR NHL [116]. Among 24 evaluable patients with CLL and 3 with NHL, all showed a lymph node reduction of more than 50% as their best response. Responses were observed in all high-risk cytogenetic subgroups of CLL. A Phase 1 study of ACP-319 combined with acalabrutinib in RR CLL is ongoing (#NCT02157324).

#### 5.4.6. Amdizalisib

Amdizalisib (HMPL-689, HUTCHMED, Hong Kong, China) is a novel PI3Kδ inhibitor [117]. In the phase study, 75 patients with CLL and other NHL had received at least one dose of HMPL-689 [127]. The ORR was 51.7%, and the median time to response (TTR) was 1.9 months. The most common TEAEs were neutropenia, increases in ALT and AST, leukopenia, hypertriglyceridaemia, pneumonia, and upper respiratory tract infection. The most common grade 3 or higher adverse events were neutropenia, pneumonia, and rash.

#### 5.4.7. SHC014748M

SHC014748M (Nanjing Sanhome Pharmaceutical, Nanjing, Jiangsu, China) is a potent, selective PI3Kδ isoform inhibitor that treats NHL and CLL/SLL [118]. SHC014748M demonstrated promising preclinical antitumor activity in B-cell NHL and CLL [119]. The Phase 1 study SHC014748M in patients with CLL and other indolent B-cell haematologic malignancies has been initiated (#NCT03588598).

#### 5.4.8. TQ-B3525

TQ-B3525 (Chia Tai Tianqing Pharmaceutical Group, Lianyungang, Jiangsu Province, China) is a selective PI3Kα/δ inhibitor. TQ-B3525 has shown promising early clinical activity and a favourable safety profile. In a phase 1 study involving 27 patients with RR lymphoma and 13 patients with advanced solid tumours, the most common all-grade adverse events were hyperglycaemia (65.0%), increased glycosylated haemoglobin (35.0%), diarrhoea (32.5%), grade ≥ 3 hyperglycaemia (10.0%), and grade ≥ 3 hypertension (3.8%) [128]. In a phase II study, TQ-B3525 was investigated in 82 patients with RR FL after at least two prior therapies. The ORR was 88.0% with 34.2% achieving CR. With a median follow-up of 13.3 months, median PFS was 18.5 months and the 24-month OS rate was estimated at 86.1% [120]. TQ-B3525 showed a favourable safety profile, with Grade 3 or higher treatment-related adverse events observed in 63 (76.8%) cases, including neutropenia (22.0%), hyperglycemia (19.5%), and diarrhoea (13.4%). DLT was grade 3 hyperglycemia, observed in three patients. Among 23 lymphoma patients, the ORR was 60.9%, and the median PFS was not reached. TQ-B3525 in patients with RR CLL/SLL is ongoing (#NCT04808570).

#### 5.4.9. Linperlisib

Linperlisib (YY-20394, Shanghai Yingli Pharmaceutical C, Shanghai, China) is a PI3Kδ inhibitor structurally distinct from idelalisib, with reduced activity against PI3Kγ. This results in a kinase inhibition profile that is more selective for Pi3kδ by nearly two orders of magnitude [121]. In a phase 1 study, 25 patients with B-cell haematological malignancies received 20–200 mg of YY-20394 daily (#NCT03757000). YY-20394 was well-tolerated with encouraging preliminary efficacy. The ORR was 64.0%, including five patients with CR, 11 with PR, and 2 with stable disease. The median PFS was 255 days. The most common drug-related adverse events included neutropenia (44.0%), pneumonia (16.0%), hyperuricemia (12.0%), lymphocytopenia (8.0%), leukopenia (8.0%), and pneumonitis (8.0%). Further development of this agent is justified.

## 6. Monoclonal Antibodies

Monoclonal antibody (mAb)-based therapeutic strategies have transformed the treatment of CLL. Rituximab, approved in 1997, was the first CD20-specific mAb authorised for treating lymphoid malignancies. Subsequently, a new generation of CD20 mAbs was developed and used for CLL treatment, both as monotherapy and, more commonly, in combination with chemotherapy or targeted drugs. Developing novel antibodies, immunotoxins, and bispecific antibodies might further enhance the efficacy of targeted therapies for CLL (Table 5) [129].

### 6.1. Novel Monoclonal Antibodies

#### 6.1.1. Belimumab

The tumour necrosis factor (TNF) family member B-cell-activating factor (BAFF) plays a crucial role in the survival of both healthy and malignant B cells. High BAFF serum levels have been shown in patients with rheumatoid arthritis and SLE related to aberrant B cell activation [130]. Moreover, elevated levels of BAFF have also been detected in patients with B-cell lymphoma. BAFF promotes the survival of malignant B-cells. It has been linked to resistance against agents such as ibrutinib and venetoclax by inhibiting apoptosis and preserving mitochondrial integrity. Belimumab (Benlysta, GSK, London, UK), an anti-BAFF antibody, was approved for treating patients with SLE and is currently being evaluated in the BeliVeR trial (#NCT05069051). This study assesses whether adding belimumab to rituximab and venetoclax proves more effective than using rituximab and venetoclax alone in patients with RR CLL.

**Table 5:** Novel monoclonal antibodies investigated in CLL.

Drug	Characteristics	Key Clinical Trials in CLL	Reference
Belimumab (Benlysta, GSK London, UK)	Recombinant human IgG-1λ mAb that inhibits BAFF	BeliVeR trial evaluated whether adding belimumab rituximab and venetoclax is more effective than using rituximab and venetoclax alone in RR CLL	#NCT05069051
Tafasitamab-cxix (MOR00208, MONJUVI, MorphoSys US Inc., Boston, MA, USA)	Humanised CD19 mAb with an engineered Fc region to enhance Fcγ receptor binding affinity	Phase 1 trial demonstrated efficacy and an acceptable safety in RR CLL [131] Phase II COSMOS study evaluated tafasitamab combined with idelalisib or venetoclax in patients with RR CLL/SLL previously treatedwith BTKi [132]	[131,132]
CAP-100 (Catapult Therapeutics, Lelystad, The Netherlands)	Humanised IgG1 mAb against CCR7, with potential immunomodulating and antineoplastic activity	Phase 1b (CAP-100-1) study evaluated safety and preliminary activity of CAP-100 in RR CLL	#NCT04704323
Cirmtuzumab (UC-961, University of California, San Diego, CA, USA)	Humanised mAb that inhibits the signalling of the ROR1	Phase 1 study of cirmtuzumab alone in RR CLL [133] Phase 1/2 study of cirmtuzumab + ibrutinib in RR MCL and RR or TN CLL [134]	[133,134] #NCT02222688, #NCT03088878
Zilovertamab vedotin (MK-2140, VLS-101, Merck & Co., Rahway, NJ, USA)	ADC that inhibits the signalling of the ROR1	Phase 1/2 study evaluated zilovertamab vedotin with ibrutinib in patients with MCL and CLL	[135] #NCT03088878

Abbreviations: ADC—antibody-drug conjugate; BAFF—B-cell activating factor; BTKi—BTK inhibitor; CCR7—chemokine receptor 7; CLL—chronic lymphocytic leukemia; CR—complete response; IKZF—IKAROS family zinc finger; mAb—monoclonal antibody; MCL—mantle cell lymphoma; ROR1—receptor tyrosine kinase-like orphan receptor 1; RR—relapsed and refractory; TN—treatment-naïve.

#### 6.1.2. Tafasitamab

Tafasitamab-cxix (MOR00208, MONJUVI, MorphoSys US Inc., Boston, MA, USA) is a humanised CD19 monoclonal antibody with an engineered Fc region to enhance Fcγ receptor binding affinity. A phase 1 trial demonstrated preliminary efficacy and an acceptable safety profile of MOR00208 in patients with RR CLL [131]. In a phase IIa study, MOR00208 monotherapy demonstrated promising clinical activity in patients with RR B-cell NHL, including DLBCL and FL, and an acceptable safety profile [136]. Responses were noted in 10 (22%) of 46 patients refractory to rituximab. In a phase II COSMOS study, tafasitamab-cxix was combined with idelalisib or venetoclax in patients with RR CLL/SLL receiving a BTKi therapy or intolerant of such therapy [132].

The combination of tafasitamab-cxix with venetoclax or idelalisib was well tolerated and showed promising antitumour activity in patients with RR CLL who had previously discontinued treatment with ibrutinib. Among the 24 patients included, 11 received idelalisib and 13 received venetoclax. The most common adverse events were anaemia, neutropenia, and infusion-related reactions. The highest ORR was 90.9% in patients receiving idelalisib and 76.9% in those receiving venetoclax. In 2020, the FDA granted accelerated approval to tafasitamab-cxix, in combination with lenalidomide, for patients with RR DLBCL not otherwise specified, arising from low-grade lymphoma, who are not eligible for autologous stem cell transplant. In 2025, approval was granted for patients with RR FL, along with lenalidomide and rituximab lymphoma.

#### 6.1.3. CAP-100

CAP-100 (Catapult Therapeutics, Lelystad, The Netherlands) is a humanised immunoglobulin G1 (IgG1) mAb against CC-chemokine receptor 7 (CCR7), with potential immunomodulating and antineoplastic properties. It blocks the ligand-binding site and signalling of CCR7 [137]. In a preclinical study, CAP-100 inhibits CCR7-induced migration, extravasation, homing, and survival in CLL samples [138,139]. CAP-100 triggers potent tumour cell killing, mediated by host immune mechanisms. CCR7 expression is down-modulated in CLL patients treated with ibrutinib and venetoclax [140]. Phase 1b of the trial (expansion phase) evaluated the safety and preliminary clinical benefit of CAP-100 monotherapy to support the design of future trials investigating CAP-100 either as monotherapy or in a combination setting with approved treatments for CLL. This phase 1b (#NCT04704323) was initiated and showed a favourable toxicity profile. CAP-100 and ibrutinib have complementary non-overlapping mechanisms of action, potentially allowing for combination therapy [141].

#### 6.1.4. ROR1 Inhibitors

ROR1 is an oncofetal protein expressed on the surface of CLL cells but is mostly absent on normal B cells [142,143,144]. For patients with CLL, self-renewing, neoplastic B cells express ROR1 in 95% of patients. High-level leukemia cell expression of ROR1 is associated with a poor prognosis. ROR1 acts as a receptor for Wnt5a, which can stimulate ROR1-dependent activation of Rho-GTPases in leukemia cells [143]. Inhibition of ROR1 signaling may have therapeutic activity in patients with CLL and other neoplasms [133]. Two mAbs that inhibit the ROR1 onco-embryonic kinase-like receptor were investigated in CLL: cirmtuzumab and zilovertamab vedotin (ZV).

##### Cirmutuzumab

Cirmtuzumab (UC-961, Pharmacyclics LLC, Sunnyvale, CA, USA/University of California, San Diego, CA, USA/California Institute for Regenerative Medicine, Oakland, CA, USA) is a humanised mAb that inhibits ROR1 signalling stemness signatures [133]. In a phase 1 study, 26 patients with RR CLL received four biweekly infusions of cirmtuzumab at doses ranging from 15 μg/kg to 20 mg/kg (#NCT02222688) [133]. Cirmtuzumab was well tolerated without dose-limiting toxicity (DLT) or serious AEs). Overall, 22 patients were evaluated for response but did not meet criteria for CR or PR. However, the median time to next treatment (TTNT) after CLL progression was 8.6 months. These results suggest that cirmtuzumab is a safe inhibitor of ROR1/Wnt5a signalling, suitable for further studies both alone and in combination with other agents. Cirmtuzumab in combination with ibrutinib was evaluated in a phase 1/2 study in patients with RR MCL and TN or RR CLL (#NCT03088878) [134]. In 17 evaluable patients with MCL, ORR was 82% and CR 41%, with response durations ranging from 8 to 28 months. In 34 evaluable patients with CLL, an ORR of 91% and a CR of 3% were achieved. The estimated 24-month PFS was 95% for RR patients and 87% for TN patients. The most common all-grade TEAEs in MCL and CLL patients included diarrhoea (41%), contusion (39%), fatigue (39%), urinary infection (31%), hypertension (25%), and arthralgia (23%) [134].

##### Zilovertamab Vedotin

Zilovertamab vedotin (MK-2140, also known as VLS-101, Merck & Co., NJ, USA, ZV) is an antibody-drug conjugate (ADC) that inhibits the signalling of the ROR1 receptor [142]. ZV consists of a fully humanised mAb (UC-961), a proteolytically cleavable amidocaproyl-valine-citrulline-para-aminobenzoate linker (mc-vc-PAB), and a monomethyl auristatin E (MMAE) cytotoxin. In preclinical studies, ZV inhibited Wnt5a-enhanced invasiveness of CLL cells. Moreover, the combination of zanubrutinib and ZV showed an additive effect in inhibiting matrigel invasiveness of CLL cells [145]. These results support the use of combined ZV and zanubrutinib in treating patients with CLL. In a phase 1 study, no significant clinical AEs were observed, and objective tumour responses were seen in 47% of heavily pretreated patients with MCL, including 4 PR and 3 CR, and in 3 of 5 patients with DLBCL (60%; one PR and two CR). However, objective responses were not observed in patients with CLL and other lymphoid malignancies. In heavily pretreated patients, ZV showed no unexpected toxicities and demonstrated evidence of antitumour activity, providing clinical proof of concept for the selective targeting of ROR1 as a potential new approach to cancer therapy (#NCT03833180) [146]. In a phase 1/2 study (#NCT03088878), ZV was evaluated in combination with ibrutinib in patients with MCL and CLL [135]. This combination was well tolerated and showed promising clinical responses in patients with MCL and CLL. For patients with R/R MCL treated with zilovertamab plus ibrutinib, the ORR was 89.3%, CRR 43%, median duration of response (DOR) was 34.1 months, and the OS rate at 25 months was 70%. In patients with RR CLL and a median of 2 previous lines of treatment, ibrutinib alone induced an ORR of 91.2% (CR rate, 8.8%). In patients treated with zilovertamab plus ibrutinib, ORR was 93.8% and CR 0%, with PFS at 24 months being 95%. For patients with *TP53* mutations/del (17p), the response rate was 100% at 42 months [135].

## 7. T-Cell Engagers

T-cell engagers are a type of antibody that simultaneously bind to T-cells and a tumour-specific antigen on B-cells, leading to their destruction. This approach is a promising therapeutic strategy for hematological malignancies, especially in RR B-cell malignancies, including CLL [147]. Bispecific antibodies (BsAb) are more advanced in development and some have been approved for treating B-cell NHL. They have been approved for RR FL (mosunetuzumab) and RR DLBCL (glofitamab). Epcoritamab, a CD3×CD20 BsAb, is approved as a single agent for RR DLBCL and FL [148,149]. Various BsAbs targeting molecules CD3 and CD20 are currently under investigation for CLL (Table 6). However, the use of BsAbs in CLL/SLL remains experimental. More than 10 trials investigating BsAbs targeting CLL are ongoing [150].

### 7.1. Epcoritamab

Epcoritamab (Tepkinly, AbbVie Inc., Chicago, IL, USA) was investigated as monotherapy in 40 patients with heavily pretreated RR CLL in the EPCORE CLL-1 study (#NCT04623541) [151]. All patients had prior BTKi therapy, and most exhibited high-risk disease characteristics. The ORR was 67%, including 33% CR. The median PFS was 12.8 months, and the median OS was not reached. Additionally, 9 out of 12 (75%) evaluable responders had undetectable MRD. The most common adverse events were cytokine release syndrome (CRS) (96%), diarrhoea (48%), peripheral oedema (48%), fatigue (43%), and injection-site reactions (43%). Kater et al. treated seven patients with RR CLL using epcoritamab administered subcutaneously. The most frequently reported TEAEs were CRS (100%), fatigue (71%), injection-site reactions (43%), and nausea (43%). Responses were observed in 3 of 5 pts [152]. The cytotoxicity of epcoritamab in CLL is enhanced by concurrent BTK or BCL-2 targeting [153]. Epcoritamab is being tested in a phase 1/2 trial (AETHER) evaluating the safety and efficacy of 2 regimens that combine *epcoritamab* and venetoclax in patients with RR CLL/SLL (#NCT05791409). The study is estimated to be completed in 2032 (#NCT04623541).

### 7.2. Mosunetuzumab

Mosunetuzumab (Lunsumio, BTCT4465A, GO29781, Roche, Basel, Switzerland) is another CD3×CD20 humanized BsAb approved for FL and investigated in patients with RR RT [154]. Fixed-duration mosunetuzumab monotherapy showed activity-induced ORR in 40% of patients and CR in 20%, with an acceptable safety profile in 20 patients with RR RT. An open-label phase 1/2 dose-escalation study is evaluating the safety, efficacy, and pharmacokinetics of escalating doses of mosumetuzumab alone and in combination with atezolizumab (anti-PD-L1) in patients with RR CLL and B-cell NHL and is ongoing (#NCT02500407). A phase 1b open-label, multicenter clinical trial is evaluating the safety, efficacy, and pharmacokinetics of mosunetuzumab alone and in combination with venetoclax in patients with RR CLL has also been initiated (#NCT05091424). The estimated completion date of the study is 2030.

### 7.3. Glofitamab

Glofitamab (Columvi, Roche, Basel, Switzerland) is a CD20×CD3 bispecific antibody that engages and redirects T cells to eliminate B cells [155]. Fixed-duration glofitamab monotherapy can lead to durable CR and has a manageable safety profile in RT patients. Carlo-Stella et al. treated 11 heavily pretreated RT patients (median of three prior therapies) with glofitamab monotherapy [156]. The ORR was 63.6% and the CR rate was 45.5%. CRS was mostly low grade, occurring in 72.7% of patients, and glofitamab-related neurological adverse events were observed in five patients. No fatal adverse events or those leading to discontinuation were reported. In another study, glofitamab is being evaluated alone or in combination with polatuzumab vedotin, pirtobrutinib, or atezolizumab as a potential treatment for RT (#NCT06043674).

### 7.4. Plamotamab

Plamotamab (XmAb-13676, Xencor, Inc., San Diego, CA, USA) is a human Fc domain-containing BsAb that binds CD3 and CD20. This agent was investigated in a phase 1 clinical trial evaluating the safety and tolerability of plamotamab in patients with CD20-expressing RR NHL (#NCT02924402) [157]. Efficacy was evaluated in 53 patients with RR NHL. The ORR was noted in 43% of them. The most common adverse event was CRS, which developed in 63% of patients. No severe or life-threatening neurotoxicity was observed.

### 7.5. GB261

GB261 (CND261, Genor Biopharma, Shanghai, China) is the first highly differentiated CD20×CD3 bispecific T cell engager designed to maintain Fc effector function, i.e., antibody-dependent cellular cytotoxicity (ADCC), antibody-dependent cellular phagocytosis (ADCP), and complement-dependent cytotoxicity (CDC) to kill the target cell [163]. Furthermore, the “imbalanced” design of GB261 incorporates de-tuned CD3 binding to reduce CRS incidence and enhance the safety features of the Fc effector function. Extensive preclinical studies have demonstrated that GB261 offers a highly advantageous balance of safety and efficacy. In an ongoing first-in-human dose-escalation study conducted in heavily pretreated B-NHL patients, GB261 exhibited a favourable safety profile, with CRS being mild, transient, and less frequent than in other CD20×CD3 BsAbs. In the first-in-human dose-escalation phase 1 study, 22 patients were available for efficacy assessment. The median follow-up duration was 4.5 months, and the ORR was 73% (16/22), including a CR rate of 45.5% (10/22) CR [158]. A continuation of this study in B-cell NHL and CLL, covering phase 2a and phase 2b, is ongoing (#NCT04923048).

### 7.6. Other T-Cell Engagers Investigated in CLL

BsAbs targeting molecules other than CD3 and CD20 are also being investigated in clinical trials for CLL.

#### 7.6.1. NVG-111

NVG111 (NovalGen, London, UK) is a humanised, first-in-class tandem scFv, ROR1xCD3 bispecific antibody that binds a unique epitope on the Frizzled domain of ROR1 and redirects T cell activity via the CD3 binder. It promotes optimal T cell interaction and efficient destruction of tumour target cells while minimising cytokine production [159]. In the first-in-human Phase 1 clinical trial of NVG111 (#NCT04763083), 12 patients with RR CLL and MCL were included, and 11 were evaluated for efficacy. Eight patients received NVG-111 combined with ibrutinib, while others received NVG-111 alone. Clinical responses were observed in six (55%) patients; the median PFS was 18.7 months. Moreover, three CLL patients achieved undetectable MRD in PB [159].

#### 7.6.2. JNJ-75348780

NJ-75348780 (Johnson & Johnson, New Brunswick, NJ, USA) is a human bispecific antibody containing two binding sites, one for the tumour-associated antigen (TAA) CD22 and one for the T-cell surface antigen CD3 [160]. CD22 is often overexpressed in B-lymphoid malignancies. A Phase 1 study of JNJ-75348780 in patients with NHL and CLL was initiated in 2020 (#NCT04540796), and the results should be available this year.

#### 7.6.3. AZD5492

AZD5492 (AstraZeneca, Cambridge, UK) is a novel, first-in-class, humanised, asymmetric trispecific monoclonal IgG1 antibody. It features two Fab binding domains to CD20, one VHH binding domain to the T-cell receptor, and one VHH binding domain to a CD8 co-receptor. Preclinical evaluation of AZD5492, a new CD8-guided T cell engager for B-cell NHL indications, shows that AZD5492 preferentially engages CD8+ T cells via CD8/TCR binding. This leads to the formation of an immunological synapse with CD20+ target cells, resulting in T-cell activation and the killing of target B cells [164]. AZD5492 kills B cells through preferential engagement of CD8+ T cells and reduced CD4+ T cell activation and, as a consequence, cytokine production. In NSG humanised mice with engrafted B-cell tumours, AZD5492 showed dose-dependent anti-tumour activity, similar to conventional bivalently linked CD20×CD3 T-cell engagers with significantly lower systemic cytokine production. In cynomolgus monkeys, AZD5492 was well tolerated and caused a significant and sustained reduction in CD19+ and CD20+ B cells in the blood and lymphoid tissues. In a phase 1/2 multicenter dose escalation and expansion study, the TITANium study, AZD5492 is being tested in patients with CD20+ mature B-cell lymphoid malignancies, including CLL (#NCT06542250) [161]. The study aims to assess the safety, efficacy, pharmacokinetics, and immunogenicity of AZD5492 (#NCT06542250).

#### 7.6.4. C312

CC312 (Cytocares, Shanghai, China) is a trispecific T-cell engager that targets the B-cell surface antigen CD19, the T-cell antigen CD3, and the T-cell co-stimulatory molecule CD28 on T-cells, which leads to redirected T-cell cytotoxicity towards CD19-positive malignant B cells [165]. Phase 1 safety study of CC312 in patients with RR CD19-positive B-cell hematologic malignancies, including CLL and MCL, was initiated in 2023 (#NCT06037018).

#### 7.6.5. Nebratamig

Nebratamig (GNC-035, Biokin Pharma, Chengdu, Sichuan, China) is an octavalent, tetra-specific T-cell engager that targets ROR1, PDL1, 4-1BB, and CD3 [162]. Nebratamig binds to CD3 T-cells to redirect them to malignant B cells expressing ROR-1. The GNC-035 can also redirect T-cell cytotoxicity towards PDL1 high-expressing cells, demonstrating its potential to convert cancer cell adaptive resistance into drug sensitivity. Additionally, the GNC-035 can engage 41-BB in a non-cytolytic manner. GNC-035 is currently being evaluated as a single agent in clinical trials enrolling patients with various ROR1-positive hematopoietic malignancies and solid tumours. An open-label, multicentre, phase Ib/II clinical trial was conducted to assess the safety, tolerability, pharmacokinetics/pharmacodynamics, and antitumour activity of nebratamig in patients with RR CLL and other haematological malignancies (#NCT05944978).

## 8. Chimeric Antigen Receptor T-Cell Therapy

Chimeric Antigen Receptor T (CAR) T-cell therapy is a therapeutic approach that involves the adoptive transfer of T cells with anti-tumour activity to the patient, inducing tumour regression [166,167,168]. In this treatment, T-cells collected from a patient are ex vivo modified to express an engineered chimeric antigen receptor against a specific antigen on tumour cells. The genetically modified CAR-T cells are expanded ex vivo and then infused into the patient, where they eliminate target neoplastic cells. The most commonly used target antigen in CLL is CD19, which is highly expressed and relatively specific for this leukaemia [147]. CD19-targeted CAR T-cells are approved for DLBCL patients [169]. In a retrospective analysis of CLL patients treated with CD19-targeted CAR-T cells, ORR was 70% and a CR rate was 30%, with a median PFS of 12 months [167]. These findings highlight the potential of CAR-T cell therapy in improving outcomes for patients with CLL and its aggressive complications [169].

Second-generation CAR T-cell therapies, such as lisocabtagene maraleucel (liso-cel; Breyanzi), are very effective treatments for B-cell neoplasms, including CLL. However, CLL’s responses to these cell-based treatments were lower than those in other indolent B-cell lymphoma diseases [170]. CAR T-cells can be particularly useful in patients with disease progression post-BTK and BCL2 inhibitors. Of the CD19 CAR T-cells currently approved for clinical use in B-cell malignancies, only brexucabtagene autoleucel (Brexu-cel), and lisocabtagene maraleucel (Liso-cel) have been investigated in CLL [159,160,161,162,163,164,165,166,167,168].

### 8.1. Lisocabtagene Maraleucel

Liso-cel,(Breyanzi, Bristol Myers Squibb, Princeton, NJ, USA) an autologous CD19-directed CAR T-cell therapy, was studied in patients with RR CLL/SLL in the TRANSCEND CLL 004 study [171,172]. The response rate was 44%, including 20% of CR. It has been recently shown that a lower disease burden correlates with a higher chance of achieving a response and that liso-cel is also effective in high-risk CLL with high-risk features [173]. CD19-directed CAR T-cell therapy can induce long-lasting responses and possibly even a cure, as shown in some patients with RR CLL. The long-term potential and clonal stability of the infused CAR T-cell were observed in CLL patients. In one study, CART cells were detectable more than ten years after infusion in two patients with sustained remission [174]. Adding ibrutinib may leverage the compound’s off-target effects, resulting in a positive overall impact on treatment outcomes. It increases the persistence of activated T cells in vivo, reduces the Treg/CD4+ T cell ratio, diminishes CLL cell immunosuppression, and enhances CD19-directed CAR T-cell expansion while decreasing CRS [175,176]. A phase 1 study administered liso-cel and ibrutinib to 19 RR CLL patients after ibrutinib failure. An 83% ORR was observed, while NGS assessed MRD negativity in BM, which was seen in 61% of patients. The 1-year PFS and OS rates were 59% and 86%, respectively [176]. The FDA has recently approved Liso-cel as the first second-generation CAR T-cell product for patients with RR CLL/SLL who have relapsed after two or more lines of therapy, including BTKis and BCL-2is. However, it has not yet been approved in Europe.

### 8.2. Brexucabtagene Autoleucel

Davids et al. evaluated brexucabtagene autoleucel (Brexu-cel, KTE-X19, Kite Pharmaceuticals, Inc., Santa Monica, CA, USA), a CD19-targeted CAR T-cell therapy, in 15 heavily pretreated patients with RR CLL in the phase 1 ZUMA-8 clinical trial [177,178]. At a median follow-up of 24.3 months, seven patients responded (47%), including one CR (7%). However, durable clinical responses were mainly limited to patients with low tumour burden. One grade 4 CRS and grade ≥ 3 neurologic events occurred in 3 patients (20%).

### 8.3. JCAR014

In the phase 1/2 study (#NCT01865617), Liang et al. treated 49 patients with JCAR014 (Juno Therapeutics, Seattle, WA, USA/Bristol-Myers Squibb/Princeton, NJ, USA), a novel CAR T-cell therapy, including 19 with concurrent ibrutinib. At a median follow-up of 79.6 months, the median PFS for all included patients was 8.9 months, and the median OS was 25.0 months (95% CI, 11.5–62.1). The 6-year PFS rate was 17.8% (95% CI, 9.7–32.8%), and the 6-year OS rate was 31.2%. A 6-year DOR was estimated at 26.4% [179].

### 8.4. New Generation of CAR T-Cell Therapies

A new generation of CAR T-cell therapy has shown promising efficacy and safety in a phase 1/2 study of heavily pretreated patients with CLL [180]. Third-generation CAR T-cell therapies mitigate the exhaustion observed with second-generation CAR T-cells by modifying the CAR vector, which enables enhanced and faster expansion responses [181]. Dergis et al. performed the first phase 1/2 trial evaluating third-generation CAR T-cell therapy (HD-CAR-1) targeting CD19 in 9 heavily pretreated patients with RR CLL and B-cell lymphoma (#NCT03676504) [169]. All patients were on BTKi and received at least one venetoclax-based treatment. HD-CAR-1 demonstrated high efficacy and exceptionally low treatment-specific toxicity. Six patients (67%) obtained a CR, including five (83%) with undetectable MRD. The 2-year PFS and OS rates were 30% and 69%, respectively. HD-CAR-1 products of responders contained significantly more CD4+ T cells than non-responders. In non-responders, a strong enrichment of effector memory-like CD8 +  T cells with high CD39 and/or CD197 expression was observed (#NCT03676504).

CAR T-cell therapy can cause acute toxicities such as CRS and immune effector cell-associated neurotoxicity syndrome (ICANS). Additionally, secondary T-cell lymphoma is a potential complication of CAR T-cell therapy [182,183].

## 9. Conclusions

Over the past decade, the treatment landscape for CLL has undergone a profound shift, transitioning from traditional chemotherapy to targeted oral therapies. BTKis and venetoclax-based regimens have significantly enhanced survival outcomes and enabled time-limited, MRD-guided treatment strategies. However, CLL remains incurable, and disease resistance—particularly in double-refractory patients—poses an emerging clinical challenge.

Novel therapeutic agents, including ncBTKis, next-generation BCL-2 inhibitors, and BTKdeg, demonstrate promising activity in heavily pretreated and high-risk groups. Furthermore, immunotherapeutic approaches such as bispecific antibodies and CAR T-cell therapy offer additional options for selected patients with refractory disease.

Future CLL management will increasingly rely on personalised treatment approaches based on genetic and molecular biomarkers, MRD status, and individual risk profiles. Ongoing clinical trials are expected to expand therapeutic options further, potentially improving long-term disease control and quality of life. Although a cure remains out of reach, the rapid development of new agents moves the field closer to achieving durable, treatment-free remission in many patients.

## Figures and Tables

**Table 1:** BTK inhibitors approved or under investigation for the treatment of CLL.

Drug	Characteristics	Selected Clinical Trials in CLL	FDA Approval for CLL	Reference
Ibrutinib (PCYC-1102, Imbruvica, Johnson & Johnson, New Brunswick, NJ, USA)	First in class covalent, irreversible BTKi	Phase 3 RESONATE study: Ibr vs. Ofa in RR CLL [16] Phase 3 RESONATE2 study: Ibr vs. Chl in TN CLL [17] Phase 3 E1912 and FLAIR studies: in TN CLL Ibru + R vs. FCR I + V vs. FCR [18,19]. Phase 3 A041202: Ibru + R vs. BR in TN CLL [20]	2014: Ibr-RR 2016: Ibr-TN 2019: Ibr + R-TN 2020: Ibru + Obi-TN	[18,19,20,21,22,23]
Acalabrutinib (ACP-196, Calquence, AstraZeneca, Cambridge, UK)	Next-generation covalent, irreversible BTKi more selective and less toxic than ibrutinib	Phase 3 ELEVATE-TN study, Acala + Obi vs. Acala vs. Chl + Obi in TN CLL [24] Phase 3 ASCEND: Acala vs. Idela + R or BR in RR CLL [25] Phase 3 ELEVATE-RR study, Acala vs. Ibru in RR CLL [26]	2019: Acala +/− Obi for CLL	[24,25,26,27]
Zanubrutinib (BGB-3111, Brukinsa, BeOne Medicines, Cambridge, MA, USA)	Next-generation covalent, irreversible BTKi with greater specificity and better bioavailability compared with ibrutinib	Phase 3 SEQUOIA study: Zanu vs. BR in TN CLL [28] Phase 3 ALPINE study: Zanu vs. Ibru in RR CLL [29]	2023: Zanu for TN CLL/SLL	[28,29]
Orelabrutinib (ICP-022, Hibruka, InnoCare, Beijing, China)	Highly selective, covalent, irreversible BTKi Greater specificity and better bioavailability compared with ibrutinib	Phase 2 study: Orelabrutinib for RR CLL [30]	Not approved by FDA, approved in China for R/R CLL/SLL	[30,31]
Tirabrutinib (Velexbru, ONO/GS-4059, Ono Pharmaceutical Co., Ltd., Osaka, Japan)	Second-generation, highly selective, covalent, irreversible oral BTKi with the ability to cross the blood–brain barrier	Phase 1 study: Tirabrutinib in RR CLL and NHL [32] Phase 2 study: Tirabrutinib + entospletinib or Tirabrutinib + entospletinib + Obi [33]	Not approved by FDA, approved in Japan to treat primary central nervous system lymphoma	[32,33]
Pirtobrutinib (LOXO-305, Jaypirca, Eli Lilly, Indianapolis, IN, USA)	Highly selective, non-covalent, reversible next-generation BTKi, inhibiting diverse BTK C481 substitution mutations	Phase 1/2 BRUIN study: RR CLL previously treated with BTKi: ORR 73.3%, most common AEs-infections (in 71.0%), bleeding (in 42.6%), and neutropenia (in 32.5%) [34]	Approved in 2023 for patients with RR previously been treated with covalent BTKi and a BCL-2	[34]
Nemtabrutinib (MK1026, Merck, Rahway, NJ, USA)	Non-covalent, reversible inhibitor of both the wild-type and the mutation C481S of BTK	Phase 1 study in R/R CLL, NHL, and WM [35]	Not approved by FDA	[35,36,37]
Rocbrutinib (LP-168, Hansoh Pharma (Lianyungang, Jiangsu, China)	Selective next-generation BTKi that reversibly targets C481 mutant BTK and irreversibly other non-C481 mutations, including T474I	Phase 1 Trial in RR CLL with Gatekeeper mutation [38]	Not approved by FDA	[38]

Abbreviations: Acala—acalabrutinib; AE—adverse event; BR—bendamustin + rituximab; BTK—Bruton tyrosine kinase, BTKi—BTK inhibitor; BCL-2—B-cell lymphoma 2; CLL—chronic lymphocytic leukemia; Chl—chlorambucil; FCR—fludarabine, cyclophosphamide, rituximab; FDA—Food and Drug Administration; FL—follicular lymphoma; Ibr—ibrutinib; I + V—ibrutinib + venetoclax; m—months; NHL—non-Hodgkin lymphoma; PLCG2—Phospholipase C gamma 2; Obi—obinutuzumab, Ofa—ofatumumab; ORR—overall response rate; OS—overall survival; PFS—progression free survival; R—rituximab RR—relapsed/refractory, SLL—small lymphocytic lymphoma; SAE—severe AE; TEAE—treatment emergent AE; TN—treatment-naïve; WM—Waldenstrom macroglobulinemia; Zanu—zanubrutinib.

**Table 2:** BTK degraders investigated in CLL.

Drug	Characteristics	Key Clinical Trials in CLL	Reference
BGB-16673 (BeOne Medicines, Cambridge, MA, USA)	Bivalent BTK degrader specifically binding to BTK and the E3 ligase	Phase 1 trial RR CLL/SLL (CaDAnCe-101): ORR 38/49 (78%), CR 2/49 (4%), AEs: fatigue, contusion, anemia, diarrhea, neutropenia, No AF, No G ≥ 3 hypertension	[60] #NCT05006716
*Bexobrutideg* (*NX-5948*; Nurix Therapeutics, Inc., San Francisco, CA, USA)	Induces BTK protein degradation by the cereblon E3 ligase without degradation of other cereblon neosubstrates	Phase 1a/b trial RR CLL/SLL: ORR 76.4%, CR 0%, AEs: contusion, thrombocytopenia, neutropenia, no AF	[61] #NCT05131022
NX-2127 (Nurix Therapeutics Inc., San Francisco, CA, USA)	Concomitant immunomodulatory activity mediating degradation of IKZF1 and IKZF3 through interactions with the cereblon E3 ubiquitin ligase complex	Phase 1a/b trial R/R CLL/SLL: ORR 9/24 (37.5), CR 0/24 (0%), SD 11/24 (46%), AEs: fatigue, neutropenia hypertension, anemia, atrial arrythmia	[62,63,64] #NCT05131022
AC676 (Accutar Biotech., Cranbury, NJ, USA)	Recruits BTK by linking a BTK ligand to the cereblon E3-ligase recruiting ligand	Phase 1, dose-escalation study in patients with relapsed/refractory B-cell malignancies (Ongoing)	[65] #NCT05780034
NRX-0492 (Nurix Therapeutics, Inc., San Francisco, CA, USA)	Selectively catalyzes ubiquitylation and proteasomal degradation of BTK	Phase 1a/1b multicenter, open-label study in patients with RR B-cell malignancies. Estimated study completion—2026	[58] #NCT04830137
HZ-Q1070 (Hangzhou HealZen Therapeutics Co., Hangzhou, China)	Novel BTK-PROTAC agents within the DaTProD^®^ platform, avoided degradation of Aiolos and Ikaros	A phase 1study in patients with RR B-cell malignancies, including CLL/SLL ongoing	[66,67] #CTR20240471
ABBV-101 (AbbVie Inc., Chicago, IL, USA)	Solely bind to and impede the catalytic domain of BTK	Phase 1, multicenter study in RR patients with B-cell NHL, including CLL	[68] #NCT05753501

Abbreviations: AE—adverse event; AF—atrial fibrillation; BTK—Bruton tyrosine kinase; CLL—chronic lymphocytic leukemia; CR—complete response; IKZF—IKAROS family zinc finger; NHL—non-Hodgkin lymphoma; ORR—overall response rate; PROTAC—Proteolysis-Targeting Chimera, RR—relapsed/refractory; SLL—small lymphocytic lymphoma.

**Table 3:** BCL-2 inhibitors approved or potentially useful for the treatment of CLL.

Drug	Characteristics	Selected Clinical Trials in CLL	FDA Approval	Reference Number
Venetoclax (formerly ABT 199, Venclyxto, AbbVie Inc., Chicago, IL, USA)	First-generation inhibitor of the apoptosis regulator BCL-2, disrupting inter-action between BCL-2 and proapoptotic proteins	Phase 3 MURANO study in RR CLL: VR vs. BR, PFS 54.7 months vs. 17.0 months, with BR. The 7-year OS 69.6% vs. 51.0%, serious AE 52.1% vs. 44.7% [70] Phase 3 CLL14 study in TN CLL: VenO vs. ChlO at median follow-up 76.4 m PFS 76.2 vs. 36.4 m, uMRD in PB 75.5% vs. 35.2%, in BM 56.9% vs. 17.1%, Grade 3 or 4 neutropenia, 52.8% vs. 48.1%, infections 17.5% vs. 15.0%, respectively [71]	2016—in monotherapy for RR CLL with del17p. 2018—in combination with rituximab for R/R CLL/SLL, 2019—in combination with obinutuzumab for TN CLL/SLL	[70,71,72]
Sonrotoclax (BGB-11417, BeOne Medicines, Cambridge, MA, USA)	Next-generation BCL2 inhibitor effective against both WT BCL-2 and several mutants including BCL2 G101V mutation-induced venetoclax resistance	Phase 1b/2 in TN CLL: Sonrotoclax + Zanu, ORR-100% (CR: 160 mg-36%, 320 mg-19%), At Median 8.5 m PFS 100%; All grade neutropenia-35%, COVID-19-23%, diarhea 23%	Not approved	[73]
Lisaftoclax (APG-2575, Ascentage Pharma, Suzhou, China)	BH3 mimetic BCL2-selective inhibitor	Phase 1 in RR CLL and NHL: ORR 63.6%, TEAEs-diarrhea (48.1%), fatigue (34.6%), nausea (30.8%), anemia and thrombocytopenia (28.8% each), neutropenia (26.9%), constipation (25.0%), vomiting (23.1%); Well tolerated up to 800 mg/day	Not approved	[74]
Surzetoclax (*ABBV-453*, Abbvie, AbbVie Inc., Chicago, IL, USA)	Next-generation BCL-2 inhibitor with high potency, selectivity and durable in vivo activity at low dose	Phase 1 Dose Escalation clinical trials in RR CLL	Not approved	[75] #NCT0629122
LOXO-338 (Loxo Oncology/Eli Lilly, Indianapolis, IN, USA)	Next-generation BCL-2 inhibitor designed to achieve selectivity for BCL-2 over Bcl-xL	Phase 1 clinical trial in patients with advanced B-cell NHL, including CLL ORR 19% and disease control 67%, TRAE-15% and were mostly grade 1 (11%) or 2 (4%); grade ≥ 3 or serious TRAE not reported	Not approved	[76] #NCT05024045
Mesutoclax (ICP-248, InnoCare Pharma, Beijing, China)	Novel, selective BCL-2 inhibitor	Phase 1 dose escalation and dose expansion study in RR CLL and MCL, ORR for CLL/SLL and RR MCL patients was 100%, including CR 14.3% for CLL/SLL and 71.4% for MCL. Most common TRAEs—neutropenia, thrombocytopenia, and upper respiratory tract infection	Not approved	[77,78] #NCT05728658

Abbreviations: AE—adverse event; BCL-2—B-cell lymphoma 2; BM—bone marrow; BR—bendamustine + rituximab; ChlO—chlorambucil + obinutuzumab; CLL—chronic lymphocytic leukemia; CR—complete response; FDA—Food and Drug Administration; m—months; NHL—non-Hodgkin lymphoma; ORR—overall response rate; OS—overall survival; PB—peripheral blood; PFS—progression-free survival, RR—relapsed/refractory; SLL—small lymphocytic lymphoma; TN—treatment naive, TRAE—treatment related AE; WT—wild type; VR—Venetoclax + rituximab; VenO—venetoclax + Obinutuzumab; uMRD—undetectable measurable residual disease; zanu—zanubrutinib.

**Table 4:** PI3K inhibitors approved or potentially effective for treating CLL.

Drug	Characteristics	Key Clinical Trials in CLL	FDA Approval	Reference
Idelalisib (GS-1101, CAL-101, Zydelig Gilead Sciences, Inc., Foster City, CA, USA)	First-in-class PI3Kδ inhibitor	Phase 3 randomised trial in patients with R/R CLL: Idela + R vs. R PFS 20.3 vs. 6.5, OS 40.6 m vs. 34.6 m most common AEs in the IDELA/R group pyrexia (40.0%), fatigue (30.9%), and diarrhea (29.1%)	2014: EMA and FDA approval for RR CLL in combination with R; 2022: voluntarily withdrawn by the developer	[102]
Duvelisib (IPI-145, INK1197, Copiktra, Secura Bio, Inc., Las Vegas, NV, USA)	Selective dual inhibitor of Pi3kδγ selective dual inhibitor of Pi3kδγ	Phase 2 DYNAMO trial in RR/CLL/SLL: ORR 68%, PR 68% Phase 3 DUO trial-duvelisib vs. Ofa in RR CLL/SLL. PFS 13.3 m vs. 9.9 months. ORR 74% vs. 45%, most common non-haematological AEs in duvelisib arm: diarrhoea (51%), pyrexia (29%), nausea (23%), cough (21%) and colitis (13%)	2018: approved for CLL after at least two therapies	[103,104,105]
Umbralisib (Ukoniq, TG Therapeutics, Morrisville, NC, USA)	Next-generation inhibitor of PI3Kδ and casein kinase-1ε (CK1ε)	Phase 2 study, Umbralisib in patients with CLL intoleratednt to prior BTKis or PI3Kδis. Median PFS 23.5 months, and ORR 44%. Most common AEs: rash (27%), arthralgia (18%), and AF (16%) Phase 3 UNITY trial: ChlO vs. umbralisib vs. ublituximab vs. umbralisib + ublituximab (U2) in TN or RR CLL. U2 well tolerated significantly improved PFS vs. ChlO	2022: FDA approval for CLL in combination with anti-CD20 antibody. Umbralisib voluntarily withdrawn for sale for approved indications by the developer	[106,107,108]
Zandelisib (PWT143, ME-401, Mei Pharma, San Diego, CA, USA)	Selective, non-covalent inhibitor of PI3Kδ	Phase 1b 89% in CLL/SLL study (ME-401-K02 study in patients with RR CLL or NHL +/− R in CLL/SLL: ORR 89%, PFS NR, Grade 3 AESI diarrhea (3.5%), colitis (3.5%), rash (2%), ALT increased (2%), and pneumonitis (2%) [104], NCT02914938	2020—FDA fast track designation for adult patients with RR FL who have receive	[109,110,111] #NCT02914938
Parsaclisib (IBI-376, Incyte/Innovent, Wilmington, DE, USA)	Highly selective, next-generation inhibitor of PI3Kδ	Parsaclisib demonstrated durable responses and a manageable safety profile in RR MZL Phase 1/2 study of parsaclisib + tafasitamab in RR CLL and RR NHL ongoing as part of a phase 1/2 study (NCT04809467)	Not approved	[112,113] #INCB050465
BGB-10188 (BeOne Medicines, Cambridge, MA, USA)	Highly selective PI3Kδ inhibitor	Phase 1/2 trial in RR CLL of BGB-10188 +/− Zanu and tislelizumab (#NCT04282018)	Not approved	[114] #NCT04282018
Tenalisib (GDC-0032, RP6530, Rhizen Pharmaceuticals SA, Basel, Switzerland)	Dual PI3K δ/γ inhibitor	Phase 2 study investigating the efficacy and safety of tenalisib in patients with RR-CLL, ongoing (#NCT04204057)	Investigational, not approved	[115] #NCT04204057
ACP-319 (AMG 319, Acerta Pharma BV, Oss, The Netherlands/AstraZeneca, Cambridge, UK)	Selective PI3Kδ inhibitor	Phase 1 study in RR-CLL and RR-NHL—ongoing [116] Phase 1 study evaluating ACP-319 + Acala and ACP-319 in RR CLL -ongoing (#NCT02157324)	Not approved	[116] #NCT02157324
Amdizalisib (HMPL-689) (HUTCHMED, Hong Kong, China)	Selective inhibitor of PI3Kδ	Phase 1b study CLL and other ORR 51.7%, median TTR—1.9 months, most common grade 3 or higher AEs—neutropenia, pneumonia, and rash	Not approved	[117]
SHC014748M (Nanjing Sanhome Pharmaceutical, Nanjing, Jiangsu, China)	Selective PI3Kδ inhibitor	Phase 1 study in patients with CLL and other indolent B-cell haematologic malignancies has been initiated (#NCT03588598).	Not approved	[118,119] #NCT03588598
TQ-B3525 (Chia Tai Tianqing Pharmaceutical Group, Lianyungang, China)	Selective PI3Kα/δ inhibitor	Phase 1 dose-escalation and expansion trial in R/R NHL and solid tumors—promising clinical activity and a favourable safety profile. Most common AEs—hyperglycaemia (65.0%), increased glycosylated haemoglobin (35.0%), diarrhoea (32.5%) (#NCT03510767)	Not approved	[120] #NCT03510767
Linperlisib (YY-20394, Yingli Pharmaceutical C, Shanghai, China)	PI3Kδ inhibitor with reduced activity against PI3Kγ	Phase 1 study in RR *B-cell NHL*: ORR 64.0%, median PFS 255 days. The most common drug-related AEs—neutropenia (44.0%), pneumonia (16.0%), hyperuricemia (12.0%), lymphocytopenia (8.0%), leukopenia (8.0%), pneumonitis (8.0%) (#NCT03757000).	Investigational, not approved	[121] #NCT03757000

Abbreviations: Acala—acalabrutinib; AE—adverse events; AF—atrial fibrilation; ALT—alanine aminotransferase; BTK—Bruton tyrosine kinase; CLL—chronic lymphocytic leukemia; EMA—European Medicines Agency; FDA—Food and Drug Administration; FL—follicular lymphoma; Idela—idelalisib; NR—not reached; m—months; PI3K—phosphatidylinositol 3-kinase; PLCG2—phospholipase C gamma 2; Obi—obinutuzumab; Ofa—ofatumumab; ORR—overall response rate; OS—overall survival; PFS—progression-free survival; PR—partial response; R—rituximab, RR—relapsed/refractory; SLL—small lymphocytic lymphoma; TN—treatment naïve; WM—Waldenstrom macroglobulinemia, Zanu—zanubrutinib.

**Table 6:** T-cell engagers examined in CLL.

Drug	Characteristics	Key Clinical Trials in CLL	Reference
Epcoritamab (Tepkinly, AbbVie Inc., Chicago, IL, USA)	BsAb CD3×CD20 approved for DLBCL, FL and high-grade B-cell Lymphoma, not approved in CLL	Phase 1b/2 study: (EPCORE™ CLL-1) in RR CLL: ORR 67%, CR 33%, median PFS-12.8 m. Most common AEs-CRS (96%), diarrhoea (48%), peripheral oedema (48%), fatigue (43%), and injection-site reactions (43%) Phase 1/2 study (AETHER) in RR CLL/SLL: Epcoritamab alone or in combination with venetoclax or pirtobrutinib in RR CLL (#NCT05791409)	[151,152,153] #NCT04623541 #NCT05791409
Mosumetuzumab (Lunsumio, BTCT4465A, GO29781 Roche, Basel, Switzerland)	BsAb CD3×CD20 humanized, approved for FL and investigated RR RT	Phase 2 study of mosumetuzumab in RR RT: ORR 40.0% and CR 20.0%, most common AE-CRS 65.0% (#NCT02500407) Phase 1 study of Mosunetuzumab and Mosunetuzumab + Venetoclax in RR CLL (NCT05091424)	[154] #NCT02500407, #NCT05091424
Glofitamab (Columvi, Roche, Basel, Switzerland)	BsAb with a novel 2:1 (CD20:CD3) format	Phase 1/2 study in RR RT: ORR 63.6% and CR 45.5%, 80% CRs ongoing for ≥24.9 months, CRS occurred in 72.7% of patients (#NCT03075696) Phase 2 study of glofitamab alone or in combination with polatuzumab vedotin, pirtobrutinib, or atezolizumab is being evaluated as a potential treatment for RT. Estimated completion 2033 (#NCT06043674)	[155,156] #NCT03075696, #NCT06043674
Plamotamab (XmAb-13676, Xencor, Inc., San Diego, CA, USA)	Human Fc domain-containing, BsAb that binds CD3 and CD20.	Phase 1 study in RR NHL: ORR 43%, most common AE-CRS 63% (#NCT02924402)	[157] #NCT02924402
GB261 (CND261, Genor Biopharma, Shanghai, China)	First BsAb CD20/CD3 designed to maintain Fc effector function	Phase 1 study in RR NHL and CLL: ORR 73%, CRR 45.5%; most common TEAEs-COVID19 infection (40.4%;) and neutropenia (31.9%), CRS 2.8% (#NCT04923048)	[158] #NCT04923048
NVG111 (NovalGen, London, UK)	Humanised, first-in-class tandem scFv, ROR1xCD3 BsAb	Phase 1 trial of NVG111 +/− ibrutinib in RR CLL and MCL. ORR was 55%, median PFS was 18.7 m, most common AEs were lethargy, headaches, nausea, vomiting and thrombocytopenia (#NCT04763083)	[159] #NCT04763083
JNJ-75348780 (Johnson & Johnson, New Brunswick, NJ, USA)	Human BsAb binding with CD22 and CD3	Phase 1 Study in RR NHL and CLL was initiated in 2020 and completed in 2025 (#NCT04540796)	[160] #NCT04540796
AZD5492 (AstraZeneca, Cambridge, UK)	Trispecific IgG1 MoAb with binding domains to CD20, one VHH to the T-cell receptor, and one VHH binding domain to a CD8 co-receptor.	Phase 1/2 (TITANium) study with CD20+ mature B-cell NHL including CLL. Estimated study completion—2028 (#NCT06542250)	[161] #NCT06542250
CC312 (Cytocares, Shanghai, China)	Trispecific T cell engager targeting CD19, CD3, T cell co-stimulatory molecule CD28 on T cells	Phase 1 study in RR CD19-positive B-cell NHL, including CLL and MCL (#NCT06037018)	#NCT06037018
Nebratamig (GNC-035, Biokin Pharma, Chengdu, Sichuan, China.)	Octavalent, tetra-specific T-cell engager targeting ROR1, PDL1, 4-1BB, and CD3	Phase 1b/2 clinical trial in R/R CLL and other haematological malignancies (#NCT05944978)	[162] #NCT05944978

Abbreviations: AE—adverse events; BsAb—Bispecific antibody; CLL—chronic lymphocytic leukemia; CRS—cytokine release syndrome; FDA—Food and Drug Administration; m—months; MCL—mantle cell lymphoma; NHL—non-Hodgkin lymphoma; PDL1—programmed death—ligand 1; PFS—progression-free survival, RR—relapsed/refractory; SLL—small lymphocytic lymphoma; SAE—severe AE; ROR1—Receptor Tyrosine Kinase-like Orphan Receptor 1; RT—Richter transformation; TEAE—treatment emergent AE.

## Data Availability

Not applicable.
